# Key Components of Prenatal Nutrition Education Interventions to Prevent Gestational Diabetes Mellitus: A Systematic Review and Meta-Analysis

**DOI:** 10.3390/nu18193303

**Published:** 2026-10-08

**Authors:** Min Gao, Yulu Zhang, Zixuan Liu, Yunqi Yang, Yadi Zhang, Shanshan Cui, Xiaxia Cai, Huanling Yu

**Affiliations:** 1 Beijing Key Laboratory of Environment and Aging, School of Public Health, Capital Medical University, Beijing 100069, China; gaomin@ccmu.edu.cn (M.G.); 13860612039@163.com (Y.Z.); liuzx1024@163.com (Z.L.); yangyunqi2021@163.com (Y.Y.); ss2cathy@163.com (S.C.); caixx1988@ccmu.edu.cn (X.C.); 2 Department of Nutrition and Food Hygiene, School of Public Health, Capital Medical University, Beijing 100069, China; 3 Department of Epidemiology, School of Public Health, Hebei University, Baoding 071002, China; yadizhang@hbu.edu.cn

**Keywords:** nutrition education interventions, pregnant women, gestational diabetes mellitus, RCTs, meta-analysis

## Abstract

**Objective:** To investigate the association between key components of prenatal nutrition education interventions and the prevention of GDM in pregnant women. **Methods**: We systematically searched PubMed, Web of Science, Embase, and the Cochrane Central Register of Controlled Trials from database inception to 18 January 2026, using search terms including “gestational diabetes mellitus,” “glycemic control,” “pregnancy,” and “nutrition interventions”; only English-language publications were eligible. Eligible studies were randomized controlled trials (RCTs) assessing prenatal nutrition education interventions and reporting gestational diabetes mellitus (GDM) incidence. Of the initial 4961 retrieved records, 29 RCTs fulfilled the selection criteria. In accordance with PRISMA guidelines, two reviewers independently performed data extraction and risk-of-bias assessment using the Cochrane RoB 2.0 tool. Given expected clinical heterogeneity, pooled risk ratios (RRs) were synthesized via a random-effects model. The primary outcome was GDM incidence, expressed as a pooled RR comparing nutrition education intervention groups with control groups. **Results**: A total of 29 RCTs comprising 17,937 pregnant women were included. The pooled analysis showed that prenatal nutrition education interventions were associated with a statistically significant reduction in GDM risk (pooled RR, 0.88 [95% CI, 0.79–0.98]; *p* = 0.02). In absolute terms, the GDM incidence was 16.01% in the intervention group vs. 17.06% in the control group. Moderate between-study heterogeneity was observed (*I*^2^ = 51.1%; *p* for heterogeneity < 0.001), largely driven by variations in dietary protocols and intervention fidelity. **Conclusions**: In this meta-analysis of 29 RCTs, prenatal nutrition education was associated with a significantly lower GDM risk. Interventions grounded in nutritional theory, delivered in hospital settings, and achieving high fidelity were associated with the largest reductions in risk; delivery by healthcare professionals, high individualization, and balanced dietary content showed favorable but statistically nonsignificant trends. These findings support the systematic adoption of these core components in routine antenatal care to mitigate the GDM burden.

## 1. Introduction

Gestational diabetes mellitus (GDM) is a globally prevalent metabolic disorder characterized by the onset of impaired glucose tolerance first detected during pregnancy and represents a significant complication of gestation. According to the International Diabetes Federation (IDF), the global prevalence reached 16.7% in 2021, with a rate of 14.8% in China that is rising alongside the increasing trend of advanced maternal age [1,2]. GDM is associated with significant short- and long-term adverse outcomes for both mothers and offspring, including obstetric complications and increased future risks of type 2 diabetes and cardiometabolic diseases [3]. Consequently, identifying effective prevention strategies is a critical public health priority.

Lifestyle modification, encompassing diet and physical activity, is recognized as a cornerstone of GDM prevention [4,5]. Pregnancy represents a unique “teachable moment” in which women are highly motivated to adopt healthier behaviors for their children’s well-being. In this context, nutrition education interventions are advocated by the World Health Organization (WHO) as a key strategy to optimize maternal nutrition and pregnancy outcomes [6,7]. Consequently, numerous randomized controlled trials (RCTs) have evaluated the efficacy of various nutrition education programs in preventing GDM. Notably, while lifestyle interventions in pregnancy have consistently shown benefits in reducing excessive gestational weight gain, their effects on GDM prevention remain variable [8,9]. This discrepancy underscores a critical gap: current clinical nutrition advice is often perceived by pregnant women as too generic, impractical, or delivered too late [10], reflecting a disconnect between theoretical recommendations and effective implementation.

As a result, robust evidence is lacking to guide policymakers in recommending optimal intervention types or target populations. Conventional study-level meta-analyses using aggregate data are further limited by heterogeneity in reported populations, interventions, and outcome definitions. It also remains unclear whether intervention effects vary by maternal characteristics or, more importantly, by specific intervention components. To date, systematic reviews have focused primarily on whether nutrition education is effective, yet they have not systematically dissected what constitutes an effective intervention. The key components associated with successful GDM prevention remain poorly understood and have yet to be identified. This gap limits the ability of clinicians and policymakers to design and implement optimal, evidence-based programs.

Therefore, the objective of this systematic review and meta-analysis is twofold: (1) to synthesize the overall effect of nutrition education interventions on GDM prevention; and (2) more critically, to identify and characterize the key components of these interventions that are associated with their effectiveness, thereby informing the development of targeted and implementable strategies in clinical practice.

## 2. Materials and Methods

### 2.1. Protocol and Guidance

This study was conducted in accordance with the Preferred Reporting Items for Systematic Reviews and Meta-Analyses (PRISMA) reporting guidelines. Details of the intended methods were documented in advance in a protocol registered in the International Prospective Register of Systematic Reviews (PROSPERO, CRD42023472979) prior to the literature search, data extraction, and analysis.

### 2.2. Literature Review

Comprehensive literature searches were conducted in the electronic databases PubMed, Embase, Web of Science, and the Cochrane Library, encompassing records from their earliest inception dates up to 18 January 2026. To maximize the retrieval of relevant studies, the search employed a combination of Medical Subject Headings (MeSH) terms and free-text terms to query titles, abstracts, and keyword fields. The core search strategy was structured as follows: (diabetes* OR glucose OR gestational diabete* OR gestational diabetes mellitus OR GDM OR glucose intolerance OR insulin resistance OR glycemic control OR pregnancy-induced diabetes) AND (pregnan* OR gravid* OR gestation* OR maternal OR antenatal) AND (lifestyle OR behavior OR diet* OR nutrition OR education OR instruction OR training OR exercise* OR physical activity) AND (intervention OR program OR modification) AND (randomized controlled trial OR random*). Additionally, the reference lists of pertinent review articles and included studies were manually screened to identify further relevant publications. The search strategy for each electronic database is presented in Table S1.

### 2.3. Study Selection: Inclusion and Exclusion Criteria

We included English-language peer-reviewed studies that assessed the association of nutrition education interventions with GDM or glycemic control in pregnant women. We defined nutrition education as instruction or education aimed at modifying food intake to enhance nutrition or dietary quality. It aims to provide evidence-based dietary guidance to pregnant individuals or groups, ensuring all nutritional requirements during pregnancy are met. This includes recommending adjustments to the intake of specific foods and offering personalized dietary recommendations. Healthcare professionals—including dietitians, physicians, nurses, and other clinical staff—deliver this counseling through various channels, such as courses, lectures, brochures, and social media platforms. In contrast, usual pregnancy health education was defined as the standard care provided during the trial, with no mention of any additional activities or interventions beyond the regular educational content.

The research questions were established using the PICOS (Population, Intervention, Comparison, Outcome, and Study Design) criteria (Table 1). We defined and included a study population consisting of pregnant women aged 18 years or older. Women who had been diagnosed with GDM or type 2 diabetes mellitus (T2DM) prior to the intervention were excluded. The intervention comprised nutrition education involving specific food intake adjustments and personalized dietary advice delivered by qualified healthcare professionals. Because most antenatal lifestyle programs combine dietary advice with physical activity, trials were eligible whenever nutrition education was a core component of the intervention, whether delivered alone or alongside physical activity; trials without a dietary or nutrition education component were excluded. Studies in which the intervention consisted solely of providing a specific nutrient (e.g., dietary fiber) were excluded. The comparison was defined as routine antenatal care or standard usual care; studies without a control group or those with a control group receiving an alternative nutritional intervention were excluded. The outcome was the incidence of GDM; studies not assessing the outcome were excluded. Only RCTs were eligible for inclusion, and non-RCT study designs were excluded.

GDM was defined according to each trial’s diagnostic criteria. In most trials, the diagnosis was made using a 75 g oral glucose tolerance test performed at 24 to 28 weeks of gestation, most commonly applying International Association of the Diabetes and Pregnancy Study Groups (IADPSG) or WHO criteria, and was ascertained from laboratory results or medical records. Because diagnostic approaches varied across trials, we accepted the case definition applied by each individual trial.

Based on the pre-established criteria, two independent reviewers (Y.Z. and M.G.) conducted the initial screening of titles and abstracts for all identified studies. The full texts of studies deemed potentially relevant or whose eligibility remained unclear after abstract screening were subsequently retrieved and subjected to a detailed eligibility assessment. Any discrepancies between the two reviewers regarding study inclusion or exclusion were adjudicated by a third independent reviewer (H.Y.).

### 2.4. Risk-of-Bias Assessment

The risk of bias and methodological quality of the studies were assessed according to the Cochrane Collaboration risk-of-bias tool 2.0 [11] by Y.Z. and M.G. The tool includes the following domains: bias due to randomization (D1), bias due to deviations from intended interventions (D2), bias due to missing data (D3), bias due to outcome measurement (D4), and bias due to selection of the reported result (D5). Each domain is rated as low risk, some concerns, or high risk of bias. The overall risk of bias for a result is classified as low if all domains are at low risk; some concerns if at least one domain has some concerns and no domain is at high risk; or high if at least one domain is at high risk.

### 2.5. Data Extraction and Organization

Two reviewers (M.G. and Y.Z.) independently conducted the extraction process. The data extracted from each eligible trial into a data extraction form encompassed the study’s objectives, subject characteristics, placebo/control information, type of nutrition education intervention, and control procedures, and cross-validation was performed to ensure accuracy. The relevant information is presented in Table S2. The Intervention Description data were extracted using the Template for Intervention Description and Replication (TIDieR) checklist, a tool developed to improve the completeness and transparency of reporting interventions in research studies, ensuring that interventions are described in enough detail for readers to replicate them in practice or future research [12]. The intervention characteristics included: (i) who (intervention providers/facilitators); (ii) tailoring (individualized plan); (iii) why (utilization of theoretical framework/model); (iv) how (application of technology and intervention modality); (v) what (intervention type, e.g., diet, intervention material and procedure, and control description); (vi) where (location where the intervention was delivered); (vii) how much (duration and frequency of sessions); and (viii) how well the intervention was delivered (fidelity and attrition). Two authors (M.G. and Y.Z.) independently coded the intervention characteristics, and disagreements were resolved by discussion (Table S3). In addition to intervention and outcome data, we extracted study-level characteristics, including country, sample size, mean maternal age, pre-pregnancy body mass index (BMI) category, race or ethnicity, education and income level, and parity, together with the diagnostic criteria and timing of GDM ascertainment and the duration and frequency of the intervention (Table 2 and Table S4). Data extracted for intervention effects included the absolute incidence of GDM, along with odds ratios (ORs) and their 95% confidence intervals (CIs). If the above-mentioned information was not reported, attempts were made to contact the corresponding authors to request the missing data. When more than one publication reported effect estimates from the same population receiving the same intervention, we reported effect estimates that encompassed a larger sample size. Univariable random-effects meta-regressions were used to examine whether mean maternal age, mean pre-pregnancy BMI, parity, and enrollment restricted to obesity explained between-study heterogeneity.

### 2.6. Statistical Analysis

Data analyses and figures were generated using the “meta” and “metafor” packages in R version 4.1.0 (R Project for Statistical Computing) [13]. Statistical significance was determined by a 2-sided *p* < 0.05. We employed the restricted maximum likelihood (REML) estimation to synthesize the results from multiple independent studies and estimate the between-study variance (I^2^) within a random-effects model. To examine the association between dietary interventions and GDM risk, all eligible trial data were pooled to estimate the summary RR (95% CI).

### 2.7. Sensitivity Analysis

A leave-one-out sensitivity analysis was performed by sequentially excluding each individual study to evaluate the robustness of the pooled estimate. Outlier studies were identified using a Galbraith plot, with standardized effect sizes beyond the 95% confidence bounds (*z* = ±1.96) considered outliers. A separate sensitivity analysis was conducted after excluding these influential studies. Cook’s distances were computed for each study, with values greater than 0.1 considered potentially influential; the pooled estimate was recalculated after excluding influential studies identified by the Galbraith plot or Cook’s distance.

### 2.8. Subgroup Analysis

To examine how intervention characteristics influenced outcomes, we conducted subgroup analyses. Studies were grouped according to key categorical variables based on TIDieR components. The pooled effect estimates were then compared across these subgroups to identify potential sources of heterogeneity.

### 2.9. Publication Bias

Publication bias was assessed using contour-enhanced funnel plot visualization, Egger’s linear regression test, Begg’s rank correlation test, and the Duval and Tweedie trim-and-fill method. The trim-and-fill method was used to impute potentially missing studies and provide a bias-corrected pooled estimate.

**Table 2:** Characteristics of the 29 included randomized clinical trials.

Study (First Author, Year)	Country	Mean Maternal Age, y (Mean ± SD)	BMI Inclusion Criterion	Education	GDM Diagnostic Criteria	Intervention Duration	Scheduled Contacts
Bruno R, 2016 [14]	Italy	31.5 ± 5.0 vs. 30.8 ± 5.5	≥25	Low/middle school 40.6%	IADPSG	9–12 wk to 36 wk	~5 dietitian contacts
Dodd JM, 2014 [15]	Australia	29.4 ± 5.5	≥25	NR	75 g OGTT	10–20 wk to 36 wk	6 contacts (face-to-face + phone)
Hui AL, 2006 [16]	Canada	26.2 ± 5.4 vs. 26.2 ± 5.7	NR	13.4 ± 3.2 vs. 11.8 ± 2.6 y	CDA 2003	20–30 wk to delivery	Weekly group sessions
Poston L, 2013 [17]	United Kingdom	30.4 ± 5.7 vs. 30.7 ± 4.9	≥30	NR	IADPSG	~19–27 wk	1:1 session + 8 weekly group sessions
Poston L, 2015 [18]	United Kingdom	30.5 ± 5.5 vs. 30.4 ± 5.6	≥30	NR	IADPSG	15–18 wk start	Interview + 8 weekly 1 h sessions
Melero V, 2020 [19]	Spain	31.7 ± 5.4 vs. 31.3 ± 5.6	NR	University 40.6% vs. 31.7%	IADPSG	12–14 wk start	2 h session, reinforced at 24–28 wk
Garmendia ML, 2020 [20]	Chile	26.1 ± 5.9	NR	Education >12 y: 22.8% vs. 20.6%	Chilean MoH	<15 wk to delivery	~6 midwife visits + PA classes
Zhang Y, 2019 [21]	China	28.1 ± 3.6 vs. 28.0 ± 3.7	≥24	NR	IADPSG	≤16 wk start	3 dietitian consultations + monthly calls
Korpi-Hyövälti E, 2011 [22]	Finland	NR	NR	College/university 18.5% vs. 44.4%	OGTT 8–12 wk	8–12 wk onward	6 nutritionist sessions
Kunath J, 2019 [23]	Germany	30.2 ± 4.4 vs. 30.4 ± 4.7	NR	University 26.2% vs. 26.8%	IADPSG	<12 wk start	3 sessions (12–16, 16–20, 30–34 wk)
Koivusalo SB, 2016 [24]	Finland	32.3 ± 4.9 vs. 32.6 ± 4.5	≥30	Higher education 30% vs. 29%	Finnish criteria	<20 wk start	3 nurse visits + 2 h dietitian session
Walsh JM, 2012 [25]	Ireland	32.0 ± 4.2	NR	NR	Two-step	~16 wk start	2 h session + reinforcement at 28 and 34 wk
Wang L, 2025 [26]	China	32.5 ± 4.4 vs. 32.5 ± 4.3	NR	College or above 83.1% vs. 81.0%	IADPSG	<14 wk start	3 dietitian consultations (13–24 wk)
Wang S, 2015 [27]	China	31.0 ± 3.8 vs. 30.3 ± 3.6	≥25	NR	IADPSG	6–8 wk start	3 group courses, reinforced at 12–13 wk
Xu J, 2023 [28]	China	31.2 ± 3.5 vs. 31.4 ± 3.6	NR	NR	IADPSG	<20 wk start	WeChat + 5 lectures + 3 dietitian assessments
H Al Wattar B, 2019 [29]	United Kingdom	31.4 ± 5.2 vs. 30.9 ± 5.2	≥30	NR	Modified IADPSG	18–28 wk	3 sessions + phone calls
Vinter CA, 2014 [30]	Denmark	29 (27–32) vs. 29 (26–31)	30–45	School ≥12 y: 74% vs. 65%	Danish (2 h ≥ 9.0 mmol/L)	10–14 wk to 35 wk	4 counseling sessions + weekly classes
Harreiter J, 2019 [31]	9 European countries	32.0 ± 5.3	≥29	Higher education 54.8%	WHO 2013 (24–28 and 35–37 wk)	<20 wk to 35–37 wk	5 coaching sessions + phone contacts
Harrison CL, 2013 [32]	Australia	32.4 ± 4.6 vs. 31.7 ± 4.5	≥25	University 46% vs. 47%	ADIPS/IADPSG	12–15 wk to 28 wk	4 sessions
Moses RG, 2014 [33]	Australia	29.9 ± 0.3	NR	NR	AADIPS/IADPSG	<20 wk to ≥34 wk	4 contacts + monthly e-mails
Kennelly MA, 2018 [34]	Ireland	32.8 ± 4.6 vs. 32.1 ± 4.2	25–39.9	Completed college 58.4% vs. 68.6%	IADPSG (28–30 wk)	10–15 wk to 34 wk	1 session + app + biweekly e-mails
Goodarzi-Khoigani M, 2017 [35]	Iran	26.6 ± 3.8 vs. 26.7 ± 4.0	NR	14.6 vs. 14.2 y	ADA	6–10 wk start	Sessions at 6–10, 18, and 26 wk
Luoto R, 2011 [36]	Finland	29.5 ± 4.8 vs. 30.0 ± 4.7	NR	University 26.8% vs. 20.6%	Finnish criteria	8–12 wk to 37 wk	PA + diet counseling visits
Chan RS, 2018 [37]	China (Hong Kong)	33.2 ± 4.4 vs. 33.1 ± 4.1	NR	Tertiary or above 61.2% vs. 52.3%	Modified WHO	≤12 wk to 24 wk	Biweekly then monthly consultations
Eslami E, 2018 [38]	Iran	25.3 ± 4.7 vs. 28.9 ± 5.6	>25	University 21.4% vs. 15.7%	75 g OGTT	16–20 wk start	Single session + SMS for 4 wk
Barquiel B, 2023 [39]	Spain	33 ± 4 vs. 33 ± 6	≥30	Higher studies 42.4% vs. 45.2%	NDDG (100 g OGTT)	~14 wk start	Single 3 h session + standard follow-up
Peacock L, 2020 [40]	United Kingdom	30.5 ± 5.5	≥30	Degree 31.3–43.5%	IADPSG	15–18 wk start	Interview + 8 weekly sessions
Ding B, 2021 [41]	China	30.6 ± 2.8 vs. 30.1 ± 2.7	≥24	NR	IADPSG	<12 wk to OGTT	3 sessions + monthly visits + WeChat
Li CL, 2021 [42]	China	37.5 ± 3.5 vs. 38.0 ± 2.5	NR	NR	ACOG 2018	First trimester to delivery	Weekly checks

Abbreviations: ADA, American Diabetes Association; ACOG, American College of Obstetricians and Gynecologists; ADIPS, Australasian Diabetes in Pregnancy Society; BMI, body mass index; CDA, Canadian Diabetes Association; GDM, gestational diabetes mellitus; IADPSG, International Association of the Diabetes and Pregnancy Study Groups; MoH, Ministry of Health; NDDG, National Diabetes Data Group; OGTT, oral glucose tolerance test; PA, physical activity.

### 2.10. Presentation of Results

The study selection process is presented using a PRISMA flow diagram. The risk-of-bias assessment results are summarized both in a table and graphically. The meta-analysis results for primary and secondary outcomes are displayed using forest plots. Publication bias was assessed using funnel plots and Egger’s test. Results of subgroup analyses and meta-regressions are presented in tables.

## 3. Results

### 3.1. Literature Search and Study Selection

**Figure 1:**
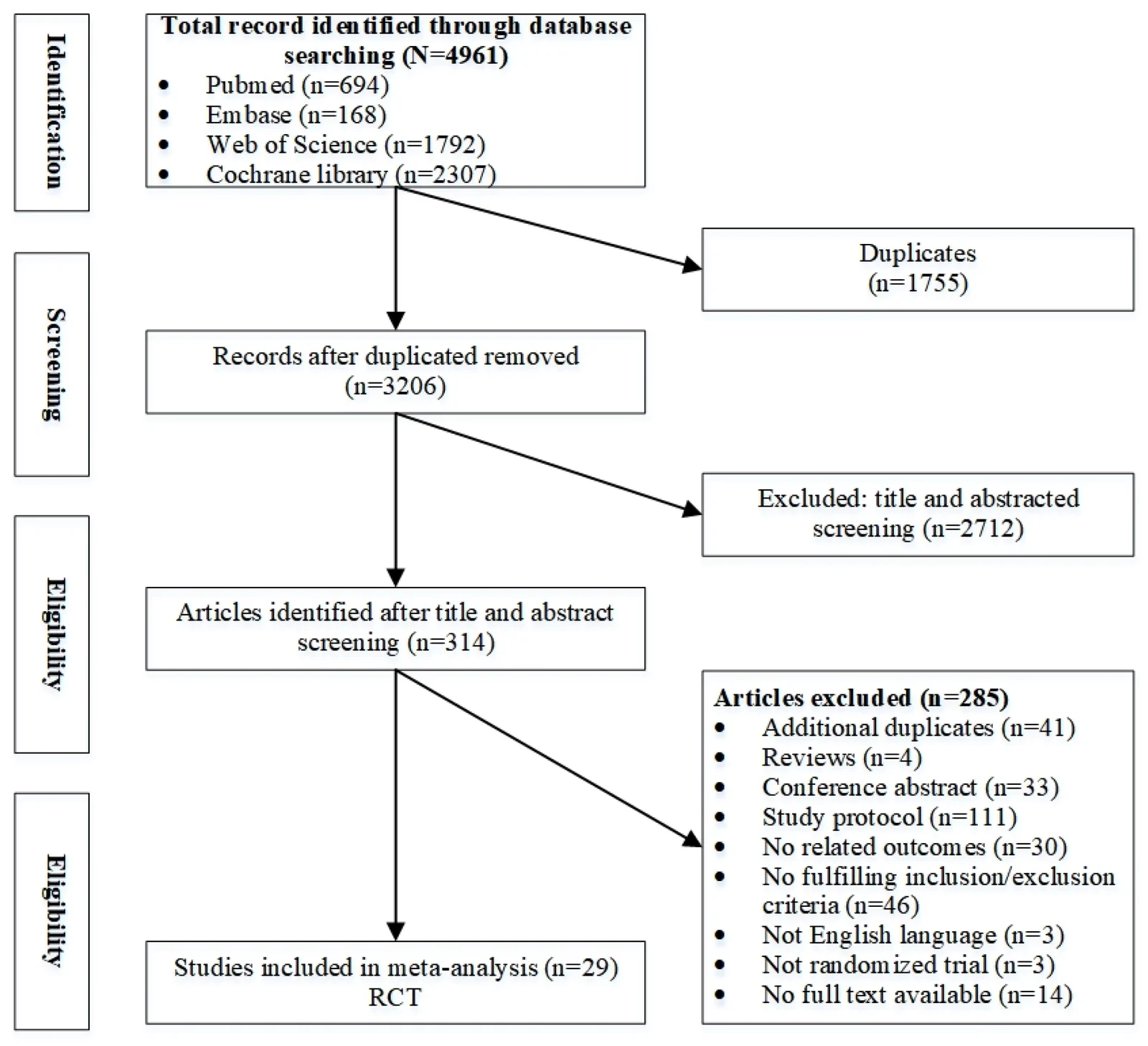
PRISMA diagram of included studies.

We identified 4961 potentially relevant studies through database searching. After removing 1755 duplicates, a total of 3206 articles remained for screening. Following title and abstract screening, 2712 records were excluded, leaving 314 articles for full-text eligibility assessment. Of these, 41 were excluded as additional duplicates, 4 were reviews, 33 were conference abstracts, 111 were study protocols, 30 did not report relevant outcomes, 46 did not meet the inclusion or exclusion criteria, 3 were not in English, 3 were not randomized controlled trials, and 14 had no full text available. Ultimately, 29 RCT studies [14,15,16,17,18,19,20,21,22,23,24,25,26,27,28,29,30,31,32,33,34,35,36,37,38,39,40,41,42] were included in the review (Figure 1).

### 3.2. Sample Characteristics and Risk of Bias

A total of 29 studies [14,15,16,17,18,19,20,21,22,23,24,25,26,27,28,29,30,31,32,33,34,35,36,37,38,39,40,41,42] with 17,937 pregnant women were finally included in the meta-analysis, of which 9274 participants were allocated to the intervention group and 8663 were allocated to the control group; all of these trials reported outcomes on GDM. Table 2 summarizes the characteristics of the included trials. The 29 trials were conducted across 12 countries, most commonly China [21,26,27,28,37,41,42] (7 trials) and the United Kingdom [17,18,29,40] (4 trials); one trial was a pan-European multicenter trial [31]. Mean maternal age ranged from 25 to 38 years. Enrollment was restricted to women with obesity (BMI ≥ 30 kg/m^2^) in 7 trials [17,18,24,29,30,39,40] and to women with overweight or obesity (BMI ≥ 24 or ≥25 kg/m^2^) in 8 others [14,15,21,27,32,34,38,41]; the remaining 14 trials applied no BMI criterion or used alternative risk-based criteria. GDM was diagnosed by a 75 g oral glucose tolerance test at 24 to 28 weeks of gestation in most trials, most often applying IADPSG or WHO 2013 thresholds (15 trials); the remainder used national or alternative criteria, including two-step procedures or the 100 g test. Intervention duration ranged from a single 3 h group session to repeated contacts extending from the first trimester to delivery. Among the 11 trials that initiated the intervention in the first trimester, 10 continued intervention contact into the second trimester. Regarding the RCTs on GDM incidence, 20 studies [15,16,20,22,23,24,26,27,28,29,30,31,32,35,36,37,38,39,41,42] aimed to promote a balanced diet, while 9 studies focused on achieving an LGI diet.

The methodological quality of the 29 randomized controlled trials was assessed using the Cochrane Risk of Bias 2 tool. Overall, 4 studies (13.8%) were judged as having a low risk of bias, 13 studies (44.8%) raised some concerns, and 12 studies (41.4%) were rated as having a high risk of bias. Among the 12 studies with an overall high risk of bias, the most common sources of bias were deviations from intended interventions (D2, n = 9) and selection of the reported result (D5, n = 7). Seven studies had a high risk in both D2 and D5 simultaneously. Six studies had a high risk of missing outcome data (D3) (Figure S1).

### 3.3. Effect of Dietary Interventions on GDM

**Figure 2:**
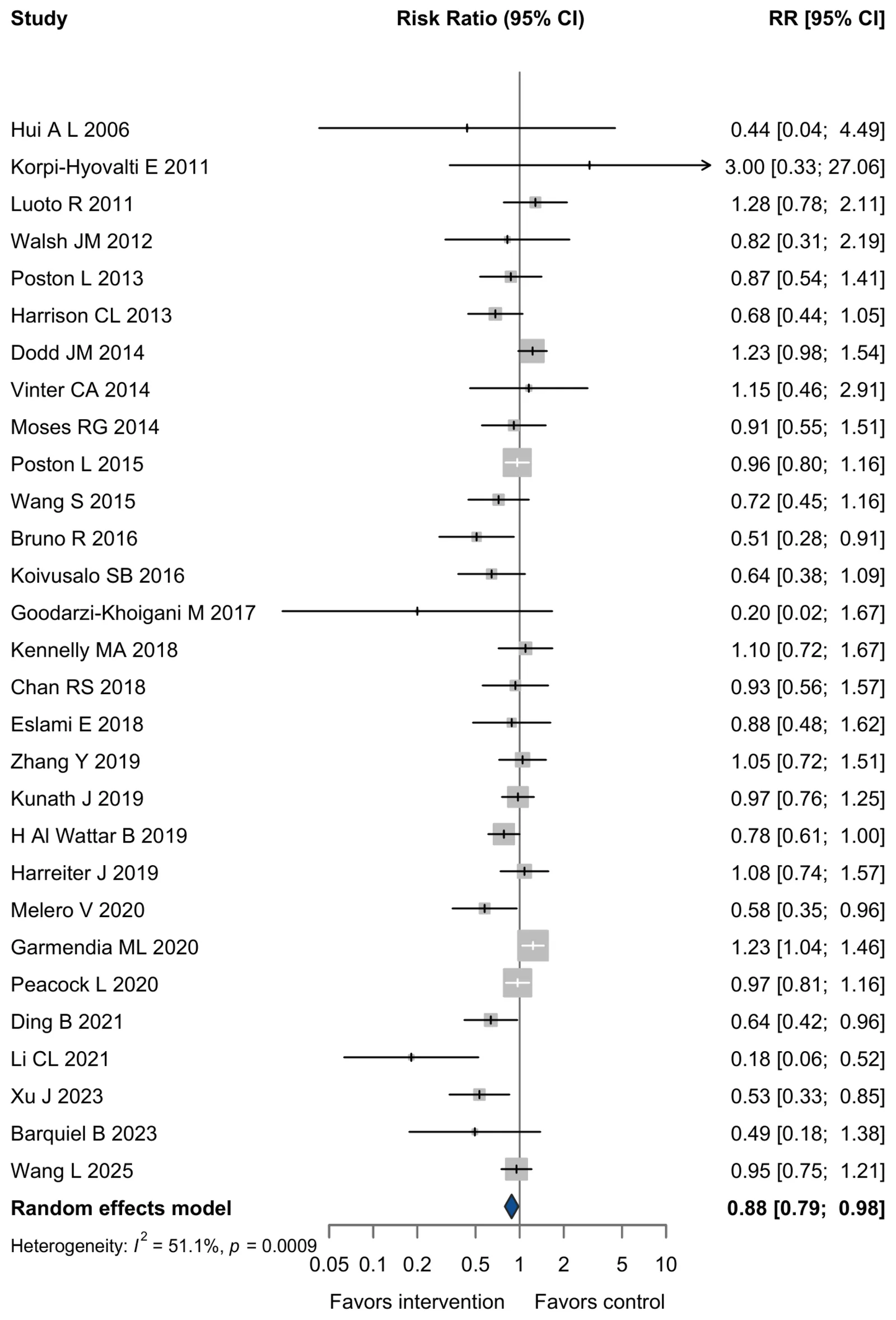
Meta-analysis of dietary interventions on GDM risk in pregnant women [14,15,16,17,18,19,20,21,22,23,24,25,26,27,28,29,30,31,32,33,34,35,36,37,38,39,40,41,42].

This meta-analysis included 29 RCTs comprising 17,937 participants (9274 in the intervention group and 8663 in the control group). The pooled analysis using a random-effects model showed that nutritional intervention was associated with a statistically significant reduction in the risk of GDM (RR, 0.88 [95% CI, 0.79–0.98]; *p* = 0.02), with moderate heterogeneity across studies (I^2^ = 51.1%; 95% CI, 25.1–68.1%; *p* for heterogeneity < 0.001) (Figure 2).

### 3.4. Subgroup Analysis Based on Research Characteristics

Subgroup analyses based on study characteristics are summarized in Table 3. For dietary intervention type, trials of low-glycemic-index (LGI) diets and trials of balanced diets yielded pooled RRs of 0.93 (95% CI, 0.84–1.03; I^2^ = 10.4%) and 0.86 (95% CI, 0.74–1.00; I^2^ = 60.5%), respectively, with no significant between-group difference (*p* = 0.43) (Figure S2). By trimester of initiation, the pooled RR was 0.78 (95% CI, 0.58–1.03; I^2^ = 72.3%) for trials starting in the first trimester and 0.92 (95% CI, 0.84–1.01; I^2^ = 18.4%) for those starting in the second trimester (between-group *p* = 0.27) (Figure S3). Stratified by overall risk of bias, trials at low risk, with some concerns, and at high risk of bias showed pooled RRs of 0.93 (95% CI, 0.71–1.22; I^2^ = 70.0%), 0.93 (95% CI, 0.85–1.01; I^2^ = 6.9%), and 0.77 (95% CI, 0.59–1.00; I^2^ = 67.5%), respectively (between-group *p* = 0.42) (Figure S4).

### 3.5. Subgroup Analysis Based on TIDieR Components

Subgroup analyses based on TIDieR components revealed varying intervention effects, as detailed in Table 3. When stratified by intervention provider, the summary relative risk (RR) for studies delivered by healthcare professionals was 0.85 (95% CI: 0.75–0.98), whereas those administered by research peers yielded an RR of 0.96 (95% CI: 0.84–1.08); the between-group difference was not statistically significant (*p* = 0.23). With respect to individualization, high-individualization interventions showed a pooled RR of 0.85 (95% CI: 0.76–0.96), compared with 1.02 (95% CI: 0.82–1.27) for low/moderate individualization, again with no significant subgroup contrast (*p* = 0.15). In contrast, a clear subgroup difference emerged for theoretical underpinnings (*p* = 0.03): interventions grounded in nutritional/physiological hypotheses produced a combined estimate of 0.81 (95% CI: 0.70–0.94), while those based on behavioral theory gave an RR of 1.00 (95% CI: 0.90–1.12). For delivery technology, traditional face-to-face approaches resulted in an estimate of 0.79 (95% CI: 0.64–0.98), and face-to-face combined with remote technology gave 0.93 (95% CI: 0.83–1.04), with no significant difference between the two (*p* = 0.19). Regarding intervention type, dietary plus physical activity yielded an RR of 0.88 (95% CI: 0.74–0.97), whereas diet-focused interventions produced an RR of 0.86 (95% CI: 0.75–1.00), and the subgroup difference was nonsignificant (*p* = 0.85). Geographic setting, however, showed a significant effect (*p* = 0.011): hospital-based studies gave an RR of 0.84 (95% CI: 0.73–0.94), community health center studies gave an RR of 1.12 (95% CI: 0.95–1.33), and mixed-setting studies gave a single estimate of 0.20 (0.02–1.67). Finally, fidelity levels also demonstrated a significant between-group difference (*p* = 0.002): high-fidelity interventions had a pooled RR of 0.76 (95% CI: 0.62–0.91), moderate-fidelity ones had an RR of 1.02 (95% CI: 0.93–1.12), and the sole study with low/moderate fidelity reported an RR of 0.51 (95% CI: 0.28–0.91).

### 3.6. Sensitivity Analysis

The leave-one-out sensitivity analysis demonstrated the robustness of the overall findings, with pooled RRs ranging from 0.87 to 0.90 after sequentially excluding individual studies (Figure S5). Based on the Galbraith plot (Figure S6), one study (Li CL 2021) [42] was identified as a statistical outlier, with a standardized effect size exceeding the 95% confidence interval bound (z = −3.00). After excluding Li CL (2021) in a sensitivity analysis, the pooled RR remained statistically significant (RR, 0.90 [95% CI, 0.81–1.00]; *p* = 0.04), with heterogeneity reduced from 51.1% to 43.6%. Cook’s distance analysis further identified four influential studies: Bruno R (2016) [14], Xu J (2023) [28], Li CL (2021) [42], and Dodd JM (2014) [15] (Cook’s D > 0.1; Figure S7).

### 3.7. Publication Bias

As shown in the contour-enhanced funnel plot (Figure S8), the distribution of studies was asymmetric, with several studies falling outside the 95% confidence region, suggesting possible publication bias. After the trim-and-fill analysis imputed eight hypothetically missing studies to adjust for the observed asymmetry, the corrected pooled estimate was attenuated and no longer statistically significant (RR, 0.97; 95% CI, 0.85–1.11; *p* = 0.67) (Figure S9).

## 4. Discussion

### 4.1. Principal Findings

This systematic review and meta-analysis aimed to investigate the efficacy of nutrition counseling to prevent GDM. To our knowledge, this is the first systematic review and meta-analysis to identify the critical components underlying successful nutrition education interventions for preventing GDM. Nutrition education interventions targeted at undernourished populations aim to address deficiencies in both energy and micronutrients. This can be achieved through the use of micronutrient supplements [43,44,45] and fortified foods or through food-based approaches [46,47]. Our meta-analysis was restricted to RCTs of food-based approaches. These interventions were selected for their potential to offer a more sustainable solution and to be more suitable for clinical application and promotion [48]. Antenatal nutrition education was associated with a 12% reduction in GDM incidence (pooled RR, 0.88; 95% CI, 0.79–0.98), and the cumulative meta-analysis corroborated this estimate. Of the TIDieR components examined, three showed statistically significant between-subgroup differences: interventions grounded in nutritional theory rather than behavioral theory (*p* = 0.03), delivered in hospitals rather than community or mixed settings (*p* = 0.009), and achieving high fidelity (*p* = 0.002). Several further components, including professional delivery, highly individualized plans, balanced dietary content, face-to-face delivery, low-intensity scheduling, and first-trimester initiation, were associated with numerically lower RRs, but the corresponding between-subgroup differences were not statistically significant.

### 4.2. Key Components of Effective Nutrition Education Interventions

Nine components characterized the more effective interventions in our subgroup analyses, although only three were supported by statistically significant between-subgroup differences. Balanced dietary goals yielded a numerically lower RR than LGI diets (0.86 vs. 0.93; *p* = 0.43), and first-trimester initiation yielded a lower RR than second-trimester initiation (0.78 vs. 0.92; *p* = 0.27), although neither contrast was significant. Delivery by medical personnel (0.85 vs. 0.96 for research peers; *p* = 0.23) and highly individualized plans (0.85 vs. 1.02; *p* = 0.15) showed trends in the expected direction. Three components rested on significant contrasts: grounding in nutritional theory (0.81 vs. 1.00; *p* = 0.03), hospital-based delivery (0.84 vs. 1.12 in community settings; *p* = 0.009), and high fidelity (0.76 vs. 1.02; *p* = 0.002). Low-intensity scheduling, defined as fewer sessions of moderate length rather than dense programming, was associated with a numerically lower RR (0.76 vs. 0.95; *p* = 0.06) and may favor feasibility and adherence. Traditional face-to-face delivery and face-to-face combined with remote technology performed comparably (0.79 vs. 0.93; *p* = 0.19), suggesting the delivery medium matters less than the content. The rationale for each component is discussed below.

### 4.3. Comparison with Other Meta-Analyses

Previous meta-analyses inconsistently noted the association of diet-based interventions with a reduced risk of GDM [49,50,51]. A meta-analysis of 40 observational studies including 30871 pregnant women by Mijatovic-Vukas J et al. [49] indicated that implementing diet-inclusive interventions represents a promising strategy for the prevention of GDM. As previously published reviews involving pregnant women have demonstrated, diet-based lifestyle interventions were associated with a reduced risk of GDM [50]. A recent meta-analysis of 104 randomized trials (35,993 women) by Allotey J et al. [51] found that the evidence is inconsistent regarding the effects of physical activity and dietary interventions during pregnancy on GDM. Inconsistent and insufficient reporting of lifestyle interventions likely explains the differences in main effects and conclusions when comparing the above studies [49,50,51]. However, current healthcare practices in many countries remain focused predominantly on the early diagnosis and treatment of GDM, with a notable absence of standardized prenatal dietary guidelines aimed at primary prevention [52]. Therefore, defining the core components of interventions and enhancing the effectiveness of nutrition education interventions are critical scientific priorities.

We conducted subgroup analyses based on the TIDieR components to identify factors associated with heterogeneity in the effectiveness of dietary interventions for GDM. Dietary interventions delivered by allied health staff using a face-to-face mode and personalized format were associated with increased efficacy. Consistent with our findings, Cheryce L. Harrison et al. [53] reached the same conclusion in their intervention study on gestational weight gain. Pregnant women with GDM generally regarded the dietary plans provided by healthcare professionals as a reliable source of information [54]. Since individual dietary practices are highly susceptible to influences such as family environment, food accessibility, sociodemographic factors, and cultural customs, individualized intervention formats may be more effective than group-based approaches by providing tailored dietary recommendations aligned with pregnant women’s specific eating habits and physiological state, thereby enhancing adherence. Interestingly, low-intensity counseling (moderate frequency and session length) showed a numerical trend toward greater benefit (*p* = 0.06, not statistically significant). Similar findings have been reported in other populations [55]. This may be explained by the fact that low-intensity programs strike a favorable balance, enabling participants to seamlessly incorporate the interventions into their daily routines, thus leading to better adherence and outcomes. Dietary education combined with physical activity (RR, 0.88) and diet-focused education (RR, 0.86) produced very similar estimates (*p* = 0.85), which suggests the nutrition education component is the shared active ingredient across these programs; the additional contribution of physical activity could not be isolated in this dataset.

Using behavioral theories was considered to be the key component that enhances the success of nutrition education interventions in previous studies [56]. However, in our findings, the type of theoretical framework, rather than theory use per se, appeared to influence efficacy. In our meta-analysis, seven RCTs employed four theories, namely the Stage Theory of Health Decision Making [15], Control Theory and Social Cognitive Theory [17,18,34], Behavior Change Theories [34], and motivational interviewing [31]. Implementing theory-based interventions has been recommended to improve behavior change effectiveness [57]. The extensiveness of theory use in the interventions may explain the findings of our meta-analysis. Most interventions demonstrated sparse use of theory, with few explicitly linking techniques to theoretical constructs or leveraging psychological concepts to inform participant selection or intervention tailoring. Of the seven theory-based trials, four showed a protective direction of effect against GDM (Poston L, 2013 [17]; Poston L, 2015 [18]; Harrison CL, 2013 [32]; Peacock L, 2020 [40]), although none reached statistical significance. Among the trials that did not reduce GDM incidence, the trial by Kennelly et al. [34] nonetheless demonstrated that the intervention based on Behavior Change Theory exerted beneficial effects on lifestyle behaviors, including reduced dietary glycemic load and increased participation in exercise. Therefore, we cannot discard the hypothesis that interventions with an extensive use of theory could turn out to be more effective in the long term, throughout life. Other RCT studies were based on nutritional hypotheses, which aim to prevent GDM through specific dietary patterns (such as a low-GI or balanced diet) or through exercise. The subgroup analysis results demonstrated that, compared with behavioral theory-based interventions, nutrition theory-based interventions were associated with greater reductions in GDM risk (between-group *p* = 0.03).

After subgroup analyses stratified by the focus of dietary intervention, we found that nutritional interventions focused on a balanced diet were more effective in preventing GDM than those focused on an LGI diet, which is consistent with the results of an earlier meta-analysis by Deng et al. [58]. A growing amount of research has focused on the effects of the LGI diet on the incidence of GDM as well. Many previous observational studies have indicated that habitual low-GI diets were related to a lower risk of GDM and better glucose homeostasis [59]. Compared to the meta-analysis by Deng et al. [58], we excluded the RCTs conducted by Geiker NRW et al. [60] and Markovic T et al. [61] due to the use of different dietary comparators (moderate-protein, moderate-glycemic index, and high-fiber, moderate-GI diets), and two RCTs conducted by Peacock L et al. (2020) [40] and Wang L et al. [26] were included. Of note, among all the RCTs included in the two aforementioned meta-analyses, only one trial conducted by Bruno R et al. (2016) [14] reported a protective effect against GDM. These RCTs failed to find a significant reduction in GDM risk by the intervention, which might partially be explained by insufficient statistical power. Compared with other RCTs, the LGI diet intervention by Bruno R et al. [14] was distinguished by its use of a detailed, personalized meal plan that incorporated participant preferences and habits, such as five daily meals and specific snack options, to enhance long-term adherence. Adherence to healthy nutrition can be considered another strong determinant of the efficacy of the intervention [14]. However, it is noteworthy that the LGI diet was associated with a poorer sense of well-being, which is likely to influence intervention adherence [62]. These meta-analysis results suggest that more attention should be paid to monitoring adherence to the diet intervention, as behavioral modification is much more difficult than merely taking a pill.

In our analysis, initiating nutritional education in the first trimester was associated with a numerically greater reduction in GDM risk (RR, 0.78 vs. 0.92), although this between-group difference did not reach statistical significance (*p* = 0.27). This finding was expected, as shown by Greaves CJ [63] et al.: the longer the intervention, the more effective it is. The timing of interventions is likely to be an important factor of benefit; most studies were initiated in the second trimester, missing opportunities to adopt appropriate behaviors in or before pregnancy to prevent GDM; shorter interventions limited the time available for the improvement of modifiable risk factors to reduce GDM. It is important to note that insulin sensitivity decreases by 56% by 34 to 36 weeks of gestation, and approximately 39% of the total decrease in insulin sensitivity occurs in the first 12–14 weeks of gestation [64]. GDM results from the short-term inability of ꞵ-cells to compensate for the physiological insulin resistance of pregnancy [65]. Therefore, interventions should be carried out in the first trimester of pregnancy to enhance the insulin sensitivity of pregnant women.

Publication bias warrants particular attention when interpreting these results. The funnel plot was asymmetric, Egger’s test indicated significant asymmetry (intercept, −1.40; t = −3.16; *p* = 0.004), and the trim-and-fill method imputed eight hypothetically missing studies, after which the pooled estimate was attenuated and no longer statistically significant (RR, 0.97; 95% CI, 0.85–1.11; *p* = 0.67). The largest reductions were also concentrated among trials at a high risk of bias (RR, 0.77 vs. 0.93 in trials with some concerns or low risk), a pattern commonly seen when smaller, lower-quality studies selectively report favorable findings. The protective association reported here should therefore be read as potentially inflated, and the component-based subgroup contrasts should be regarded as hypothesis-generating.

In exploratory meta-regressions, mean pre-pregnancy BMI, the proportion of nulliparous women, and enrollment restricted to obesity did not explain between-study heterogeneity (Table S5). Mean maternal age did: each additional year of mean age corresponded to a 7% greater relative reduction in GDM risk (β = −0.070 per year; 95% CI, −0.110 to −0.031; *p* < 0.001), and this single covariate accounted for 73% of the between-study variance. The association was not driven by any individual trial: in leave-one-out analyses, the coefficient remained statistically significant after excluding each trial in turn (all *p* ≤ 0.019), including the oldest cohort (Li CL et al., 2021) [42] and the three other trials identified as influential in the Cook’s distance analysis.

### 4.4. Strengths and Limitations

A key strength of this study is that it represents the first systematic review and meta-analysis to identify the critical components underlying successful nutrition education interventions for preventing GDM. This meta-analysis of 29 randomized controlled trials (N = 17,937) provides robust and reliable evidence that structured nutrition education interventions significantly reduce the risk of GDM and improve glycemic control in pregnant women. Our evaluation was guided by the TIDieR framework, a rigorous and widely accepted tool for characterizing intervention components. The inclusion of usual care as the comparator group enhances the generalizability of our findings to clinical settings. Furthermore, we restricted our analysis to RCTs of food-based approaches, which hold greater potential for sustainability and are more readily applicable in clinical practice and health promotion.

This study also has several limitations. First, 25 of the 29 trials were rated as having some concerns or a high risk of bias, and reporting of quality assurance measures such as adherence and fidelity was generally poor. Second, because the included studies were not reported in accordance with the TIDieR framework, the extraction of certain intervention components required subjective interpretation, and limited information on session duration, tailoring, and adherence constrained our ability to assess the associations between these specific components and intervention effects. Third, most interventions combined nutrition education with physical activity, and the independent contribution of the dietary component could not be isolated. Fourth, publication bias may be present: after trim-and-fill adjustment, the pooled estimate was no longer statistically significant, suggesting that the protective association may be inflated. Finally, most interventions made only limited use of behavioral theory, with few explicitly linking intervention techniques to theoretical constructs.

## 5. Conclusions

Across 29 randomized controlled trials, prenatal nutrition education was associated with a significantly lower incidence of GDM. Subgroup analyses supported three intervention components as being most strongly associated with benefit: content grounded in nutritional theory, delivery in hospital settings, and high fidelity. Balanced dietary goals, first-trimester initiation, healthcare professional delivery, individualization, and low-intensity scheduling showed favorable trends, but the corresponding between-subgroup differences were not statistically significant. Because trim-and-fill adjustment attenuated the pooled estimate to non-significance, and because most trials raised some concerns or were at high risk of bias, these findings require confirmation in large pragmatic trials that monitor fidelity and standardize GDM ascertainment. Future research should prioritize early intervention, structured individualization, and sustained adherence monitoring and should evaluate the proposed components across diverse antenatal care settings.

## Figures and Tables

**Table 1:** PICOS criteria for inclusion of studies.

Parameter	Inclusion Criteria	Exclusion Criteria
Population	Pregnant women aged 18 years or older	Participants who had been diagnosed with GDM or T2DM before the intervention
Intervention	Includes recommending specific food intake adjustments and providing personalized food-based solutions by healthcare professionals.	Nutrients such as dietary fiber supplementation
Comparison	Usual pregnancy health education	No control or placebo group
Outcome	GDM ^1^	Not assessing the major outcome
Study design	RCT ^2^	Non-RCT
Research questions	What are the key components for the success of prenatal nutrition education interventions?	

^1^ Gestational diabetes mellitus; ^2^ randomized controlled trial.

**Table 3:** Subgroup analyses of the effect of nutritional interventions on GDM ^1^ risk.

Subgroup	k	RR (95%CI)	*p*-Value	*I^2^*	*p*
All 29 RCTs	29	0.88(0.79–0.98)	0.02	51.1%	
**Diet type**					0.43
LGI^2^diets	9	0.93(0.84–1.03)	0.17	10.4%	
Balanced diets	20	0.86(0.74–1.00)	0.06	60.5%	
**Trimester**					0.27
First trimester	11	0.78(0.58–1.03)	0.09	72.3%	
Second trimester	18	0.92(0.84–1.01)	0.10	18.4%	
**Risk of bias**					0.42
Low	4	0.93(0.71–1.22)	0.62	70.0%	
Some concerns	13	0.93(0.85–1.01)	0.10	6.9%	
High	12	0.77(0.59–1.00)	0.05	67.5%	
**Provider type**					0.23
Healthcare professionals	25	0.85(0.75–0.98)	0.02	57.9%	
Research peers	4	0.96(0.84–1.08)	0.47	0.0%	
**Individualized approach**					0.15
High	24	0.85(0.76–0.96)	0.009	48.8%	
Low/moderate	5	1.02(0.82–1.27)	0.86	36.0%	
**Theory basis**					0.03
Nutritional theory	22	0.81(0.70–0.94)	0.007	56.3%	
Behavioral theory	7	1.00(0.90–1.12)	0.97	15.5%	
**Intervention type**					0.85
Dietary + physical activity	21	0.88(0.77–1.01)	0.06	57.6%	
Dietary as main approach	8	0.86(0.75–1.00)	0.05	11.2%	
**Geographic location**					0.009
Hospital	22	0.84(0.75–0.94)	0.004	48.4%	
Community health center	6	1.12(0.95–1.33)	0.18	0.0%	
Mixed	1	0.20(0.02–1.67)	0.14	—	
**Technology mode**					0.19
Traditional face-to-face	16	0.79(0.64–0.98)	0.03	61.7%	
Face-to-face + remote	13	0.93(0.83–1.04)	0.21	33.9%	
**Intensity**					0.06
Moderate	15	0.95(0.86–1.06)	0.39	27.6%	
Low	14	0.76(0.62–0.94)	0.01	65.5%	
**Fidelity**					0.002
Low/moderate	1	0.51(0.28–0.91)	0.02	—	
High	12	0.76(0.62–0.91)	0.004	63.0%	
Moderate	16	1.02(0.93–1.12)	0.69	0.0%	

^1^ Gestational diabetes mellitus; ^2^ low glycemic index.

## Data Availability

All relevant data are within the published studies included in this systematic review.

## Supplementary Materials

The following supporting information can be downloaded at https://www.mdpi.com/article/10.3390/nu18193303/s1, Table S1. Search strategies used in PubMed, Embase, Web of Science, and the Cochrane Library. Table S2. Characteristics of studies included in the meta-analysis of RCTs of prenatal nutrition education interventions to prevent GDM. Table S3. The TIDieR checklist of studies included in the meta-analysis of RCTs of prenatal nutrition education interventions to prevent GDM. Table S4. Sociodemographic characteristics in the 29 included trials. Table S5. Univariable random-effects meta-regressions of study-level characteristics on GDM risk. Figure S1. Traffic light plot summarizing the risk-of-bias judgments across included studies according to ROB 2.0. Figure S2. Meta-analysis of subgroup analyses stratified by dietary intervention type. Figure S3. Meta-analysis of subgroup analyses stratified by trimester of intervention initiation. Figure S4. Meta-analysis of subgroup analyses stratified by risk of bias. Figure S5. The leave-one-out sensitivity analysis of included RCTs. Figure S6. The Galbraith plot of included RCTs. Figure S7. Cook’s distances for each included study in the random-effects meta-analysis. Figure S8. The contour-enhanced funnel plot of included RCTs. Figure S9. The trim-and-fill funnel plot of included RCTs.

## References

[1] Magliano D.J., Boyko E.J. (2021). IDF DIABETES ATLAS.

[2] Gao C., Sun X., Lu L., Liu F., Yuan J. (2019). Prevalence of gestational diabetes mellitus in mainland China: A systematic review and meta-analysis. J. Diabetes Investig..

[3] Sweeting A., Wong J., Murphy H.R., Ross G.P. (2022). A Clinical Update on Gestational Diabetes Mellitus. Endocr. Rev..

[4] Moholdt T. (2023). Diet, Exercise and Gestational Diabetes Mellitus. Nutrients.

[5] Rasmussen L., Poulsen C.W., Kampmann U., Smedegaard S.B., Ovesen P.G., Fuglsang J. (2020). Diet and Healthy Lifestyle in the Management of Gestational Diabetes Mellitus. Nutrients.

[6] WHO (2016). Recommendations on Antenatal Care for a Positive Pregnancy Experience.

[7] Lucas C., Charlton K.E., Yeatman H. (2014). Nutrition Advice During Pregnancy: Do Women Receive it and Can Health Professionals Provide it?. Matern. Child Health J..

[8] Song C., Li J., Leng J., Ma R.C., Yang X. (2016). Lifestyle intervention can reduce the risk of gestational diabetes: A meta-analysis of randomized controlled trials. Obes. Rev..

[9] Tieu J., Shepherd E., Middleton P., Crowther C.A. (2017). Dietary advice interventions in pregnancy for preventing gestational diabetes mellitus. Cochrane Database Syst. Rev..

[10] de Jersey S.J., Nicholson J.M., Callaway L.K., Daniels L.A. (2013). An observational study of nutrition and physical activity behaviours, knowledge, and advice in pregnancy. BMC Pregnancy Childbirth.

[11] Sterne J.A.C., Savovic J., Page M.J., Elbers R.G., Blencowe N.S., Boutron I., Cates C.J., Cheng H.Y., Corbett M.S., Eldridge S.M. (2019). RoB 2: A revised tool for assessing risk of bias in randomised trials. BMJ.

[12] Hoffmann T.C., Glasziou P.P., Boutron I., Milne R., Perera R., Moher D., Altman D.G., Barbour V., Macdonald H., Johnston M. (2014). Better reporting of interventions: Template for intervention description and replication (TIDieR) checklist and guide. BMJ.

[13] Balduzzi S., Rucker G., Schwarzer G. (2019). How to perform a meta-analysis with R: A practical tutorial. BMJ Ment. Health.

[14] Bruno R., Petrella E., Bertarini V., Pedrielli G., Neri I., Facchinetti F. (2017). Adherence to a lifestyle programme in overweight/obese pregnant women and effect on gestational diabetes mellitus: A randomized controlled trial. Matern. Child Nutr..

[15] Dodd J.M., Turnbull D., McPhee A.J., Deussen A.R., Grivell R.M., Yelland L.N., Crowther C.A., Wittert G., Owens J.A., Robinson J.S. (2014). Antenatal lifestyle advice for women who are overweight or obese: LIMIT randomised trial. BMJ.

[16] Hui A.L., Ludwig S.M., Gardiner P., Sevenhuysen G., Murray R., Morris M., Shen G.X. (2006). Community-based exercise and dietary intervention during pregnancy: A pilot study. Can. J. Diabetes.

[17] Poston L., Briley A.L., Barr S., Bell R., Croker H., Coxon K., Essex H.N., Hunt C., Hayes L., Howard L.M. (2013). Developing a complex intervention for diet and activity behaviour change in obese pregnant women (the UPBEAT trial); assessment of behavioural change and process evaluation in a pilot randomised controlled trial. BMC Pregnancy Childbirth.

[18] Poston L., Bell R., Croker H., Flynn A.C., Godfrey K.M., Goff L., Hayes L., Khazaezadeh N., Nelson S.M., Oteng-Ntim E. (2015). Effect of a behavioural intervention in obese pregnant women (the UPBEAT study): A multicentre, randomised controlled trial. Lancet Diabetes Endocrinol..

[19] Melero V., Garcia de la Torre N., Assaf-Balut C., Jimenez I., Del Valle L., Duran A., Bordiu E., Valerio J.J., Herraiz M.A., Izquierdo N. (2020). Effect of a Mediterranean Diet-Based Nutritional Intervention on the Risk of Developing Gestational Diabetes Mellitus and Other Maternal-Fetal Adverse Events in Hispanic Women Residents in Spain. Nutrients.

[20] Garmendia M.L., Corvalan C., Araya M., Casanello P., Kusanovic J.P., Uauy R. (2020). Effectiveness of a normative nutrition intervention in Chilean pregnant women on maternal and neonatal outcomes: The CHiMINCs study. Am. J. Clin. Nutr..

[21] Zhang Y., Wang L., Yang W., Niu D., Li C., Wang L., Gu P., Xia Y., Shen Y., Yan J. (2019). Effectiveness of Low Glycemic Index Diet Consultations Through a Diet Glycemic Assessment App Tool on Maternal and Neonatal Insulin Resistance: A Randomized Controlled Trial. JMIR mHealth uHealth.

[22] Korpi-Hyovalti E., Schwab U., Laaksonen D.E., Linjama H., Heinonen S., Niskanen L. (2012). Effect of intensive counselling on the quality of dietary fats in pregnant women at high risk of gestational diabetes mellitus. Br. J. Nutr..

[23] Kunath J., Gunther J., Rauh K., Hoffmann J., Stecher L., Rosenfeld E., Kick L., Ulm K., Hauner H. (2019). Effects of a lifestyle intervention during pregnancy to prevent excessive gestational weight gain in routine care—The cluster-randomised GeliS trial. BMC Med..

[24] Koivusalo S.B., Rono K., Klemetti M.M., Roine R.P., Lindstrom J., Erkkola M., Kaaja R.J., Poyhonen-Alho M., Tiitinen A., Huvinen E. (2016). Gestational Diabetes Mellitus Can Be Prevented by Lifestyle Intervention: The Finnish Gestational Diabetes Prevention Study (RADIEL): A Randomized Controlled Trial. Diabetes Care.

[25] Walsh J.M., McAuliffe F.M. (2015). Impact of maternal nutrition on pregnancy outcome--does it matter what pregnant women eat?. Best Pract. Res. Clin. Obstet. Gynaecol..

[26] Wang L., Wang X., Zhang R., Sun W., Zhang C., Zhang C., Qin G., Peng J., Li H., Fan J. (2025). Individualized nutritional intervention compared with routine care for gestational diabetes prevention: A randomized controlled trial. Am. J. Clin. Nutr..

[27] Wang S., Ma J.M., Yang H.X. (2015). Lifestyle intervention for gestational diabetes mellitus prevention: A cluster-randomized controlled study. Chronic Dis. Transl. Med..

[28] Xu J., Lin X., Fang Y., Cui J., Li Z., Yu F., Tian L., Guo H., Lu X., Ding J. (2023). Lifestyle interventions to prevent adverse pregnancy outcomes in women at high risk for gestational diabetes mellitus: A randomized controlled trial. Front. Immunol..

[29] Al Wattar B.H., Dodds J., Placzek A., Beresford L., Spyreli E., Moore A., Gonzalez Carreras F.J., Austin F., Murugesu N., Roseboom T.J. (2019). Mediterranean-style diet in pregnant women with metabolic risk factors (ESTEEM): A pragmatic multicentre randomised trial. PLoS Med..

[30] Vinter C.A., Jorgensen J.S., Ovesen P., Beck-Nielsen H., Skytthe A., Jensen D.M. (2014). Metabolic effects of lifestyle intervention in obese pregnant women. Results from the randomized controlled trial ‘Lifestyle in Pregnancy’ (LiP). Diabet. Med..

[31] Harreiter J., Simmons D., Desoye G., Corcoy R., Adelantado J.M., Devlieger R., Galjaard S., Damm P., Mathiesen E.R., Jensen D.M. (2019). Nutritional Lifestyle Intervention in Obese Pregnant Women, Including Lower Carbohydrate Intake, Is Associated With Increased Maternal Free Fatty Acids, 3-beta-Hydroxybutyrate, and Fasting Glucose Concentrations: A Secondary Factorial Analysis of the European Multicenter, Randomized Controlled DALI Lifestyle Intervention Trial. Diabetes Care.

[32] Harrison C.L., Lombard C.B., Strauss B.J., Teede H.J. (2013). Optimizing healthy gestational weight gain in women at high risk of gestational diabetes: A randomized controlled trial. Obesity.

[33] Moses R.G., Casey S.A., Quinn E.G., Cleary J.M., Tapsell L.C., Milosavljevic M., Petocz P., Brand-Miller J.C. (2014). Pregnancy and Glycemic Index Outcomes study: Effects of low glycemic index compared with conventional dietary advice on selected pregnancy outcomes. Am. J. Clin. Nutr..

[34] Kennelly M.A., Ainscough K., Lindsay K.L., O’Sullivan E., Gibney E.R., McCarthy M., Segurado R., DeVito G., Maguire O., Smith T. (2018). Pregnancy Exercise and Nutrition With Smartphone Application Support: A Randomized Controlled Trial. Obstet. Gynecol..

[35] Goodarzi-Khoigani M., Mazloomy Mahmoodabad S.S., Baghiani Moghadam M.H., Nadjarzadeh A., Mardanian F., Fallahzadeh H., Dadkhah-Tirani A. (2017). Prevention of Insulin Resistance by Dietary Intervention among Pregnant Mothers: A Randomized Controlled Trial. Int. J. Prev. Med..

[36] Luoto R., Kinnunen T.I., Aittasalo M., Kolu P., Raitanen J., Ojala K., Mansikkamaki K., Lamberg S., Vasankari T., Komulainen T. (2011). Primary prevention of gestational diabetes mellitus and large-for-gestational-age newborns by lifestyle counseling: A cluster-randomized controlled trial. PLoS Med..

[37] Chan R.S., Tam W.H., Ho I.C., Kwan M.W., Li L.S., Sea M.M., Woo J. (2018). Randomized trial examining effectiveness of lifestyle intervention in reducing gestational diabetes in high risk Chinese pregnant women in Hong Kong. Sci. Rep..

[38] Eslami E., Charandabi S.M.A., Khalili A.F., Jafarabadi M.A., Mirghafourvand M. (2018). The Effect of a Lifestyle-Based Training Package on Weight Gain and Frequency of Gestational Diabetes in Obese and Overweight Pregnant Females. Iran. Red Crescent Med. J..

[39] Barquiel B., Calvo M., Moreno-Dominguez O., Martinez-Sanchez N., Muner M., Bedate M.F., Delgado M., Lopez S., Hillman N., Gonzalez N. (2023). The PREDG study: A randomised controlled trial testing whether an educational intervention can prevent gestational weight gain in women with obesity. Clin. Nutr. ESPEN.

[40] Peacock L., Seed P.T., Dalrymple K.V., White S.L., Poston L., Flynn A.C. (2020). The UK Pregnancies Better Eating and Activity Trial (UPBEAT); Pregnancy Outcomes and Health Behaviours by Obesity Class. Int. J. Environ. Res. Public Health.

[41] Ding B., Gou B., Guan H., Wang J., Bi Y., Hong Z. (2021). WeChat-assisted dietary and exercise intervention for prevention of gestational diabetes mellitus in overweight/obese pregnant women: A two-arm randomized clinical trial. Arch. Gynecol. Obstet..

[42] Li C.L., Wang Y.H., Wang J.L., Zhang P., Sun Y. (2021). Effect of individualized medical nutrition guidance on pregnancy outcomes in older pregnant women. J. Int. Med. Res..

[43] Bhutta Z.A., Das J.K., Rizvi A., Gaffey M.F., Walker N., Horton S., Webb P., Lartey A., Black R.E., The Lancet Nutrition Interventions Review Group, the Maternal and Child Nutrition Study Group (2013). Evidence-based interventions for improvement of maternal and child nutrition: What can be done and at what cost?. Lancet.

[44] Gomes F., Bourassa M.W., Adu-Afarwuah S., Ajello C., Bhutta Z.A., Black R., Catarino E., Chowdhury R., Dalmiya N., Dwarkanath P. (2020). Setting research priorities on multiple micronutrient supplementation in pregnancy. Ann. N. Y. Acad. Sci..

[45] Pena-Rosas J.P., De-Regil L.M., Rogers L.M., Bopardikar A., Panisset U. (2012). Translating research into action: WHO evidence-informed guidelines for safe and effective micronutrient interventions. J. Nutr..

[46] Branca F., Demaio A., Udomkesmalee E., Baker P., Aguayo V.M., Barquera S., Dain K., Keir L., Lartey A., Mugambi G. (2020). A new nutrition manifesto for a new nutrition reality. Lancet.

[47] Demaio A.R., Branca F. (2018). Decade of action on nutrition: Our window to act on the double burden of malnutrition. BMJ Glob. Health.

[48] Katenga-Kaunda L.Z., Iversen P.O., Kamudoni P.R., Holmboe-Ottesen G., Fjeld H.E. (2022). Food-based nutrition counselling and education intervention for improved diets of pregnant women in rural Malawi: A qualitative study of factors influencing dietary behaviour change. Public Health Nutr..

[49] Mijatovic-Vukas J., Capling L., Cheng S., Stamatakis E., Louie J., Cheung N.W., Markovic T., Ross G., Senior A., Brand-Miller J.C. (2018). Associations of Diet and Physical Activity with Risk for Gestational Diabetes Mellitus: A Systematic Review and Meta-Analysis. Nutrients.

[50] Teede H.J., Bailey C., Moran L.J., Bahri Khomami M., Enticott J., Ranasinha S., Rogozinska E., Skouteris H., Boyle J.A., Thangaratinam S. (2022). Association of Antenatal Diet and Physical Activity-Based Interventions With Gestational Weight Gain and Pregnancy Outcomes: A Systematic Review and Meta-analysis. JAMA Intern. Med..

[51] Allotey J., Coomar D., Ensor J., Ruiz-Calvo G., Boath A., Ogwulu C.O., Monahan M., Kabeya V., Zheng M., McNeill R. (2026). Effects of lifestyle interventions in pregnancy on gestational diabetes: Individual participant data and network meta-analysis. BMJ.

[52] Simmons D., Gupta Y., Hernandez T.L., Levitt N., van Poppel M., Yang X., Zarowsky C., Backman H., Feghali M., Nielsen K.K. (2024). Call to action for a life course approach. Lancet.

[53] Harrison C.L., Bahri Khomami M., Enticott J., Thangaratinam S., Rogozinska E., Teede H.J. (2023). Key Components of Antenatal Lifestyle Interventions to Optimize Gestational Weight Gain: Secondary Analysis of a Systematic Review. JAMA Netw. Open.

[54] Jia C.L., Wang L.J., Li L.H., Lu Y.J., Yang Y. (2025). Factors Influencing Adherence to Dietary Interventions Among Patients with Gestational Diabetes Mellitus in China: A Qualitative Study Based on the COM-B Model. J. Multidiscip. Healthc..

[55] Low J.H.M., Toh D.W.K., Ng M.T.T., Fam J., Kua E.H., Kim J.E. (2021). A Systematic Review and Meta-Analysis of the Impact of Different Intensity of Dietary Counselling on Cardiometabolic Health in Middle-Aged and Older Adults. Nutrients.

[56] Murimi M.W., Kanyi M., Mupfudze T., Amin M.R., Mbogori T., Aldubayan K. (2017). Factors Influencing Efficacy of Nutrition Education Interventions: A Systematic Review. J. Nutr. Educ. Behav..

[57] Glanz K., Bishop D.B. (2010). The role of behavioral science theory in development and implementation of public health interventions. Annu. Rev. Public Health.

[58] Deng Y., Yu J., Tao A., Liu J., Wang Q., Cao Y., Han S., Xu X., Yan X., Fang X. (2023). Effect of low-glycemic index diet advice on pregnant outcomes in women with elevated risk of gestational diabetes mellitus: A meta-analysis of randomized controlled trails. Clin. Nutr. ESPEN.

[59] Zhang X., Gong Y., Della Corte K., Yu D., Xue H., Shan S., Tian G., Liang Y., Zhang J., He F. (2021). Relevance of dietary glycemic index, glycemic load and fiber intake before and during pregnancy for the risk of gestational diabetes mellitus and maternal glucose homeostasis. Clin. Nutr..

[60] Geiker N.R.W., Magkos F., Zingenberg H., Svare J., Chabanova E., Thomsen H.S., Ritz C., Astrup A. (2022). A high-protein low-glycemic index diet attenuates gestational weight gain in pregnant women with obesity: The “An optimized programming of healthy children” (APPROACH) randomized controlled trial. Am. J. Clin. Nutr..

[61] Markovic T.P., Muirhead R., Overs S., Ross G.P., Louie J.C., Kizirian N., Denyer G., Petocz P., Hyett J., Brand-Miller J.C. (2016). Randomized Controlled Trial Investigating the Effects of a Low-Glycemic Index Diet on Pregnancy Outcomes in Women at High Risk of Gestational Diabetes Mellitus: The GI Baby 3 Study. Diabetes Care.

[62] Halperin I.J., Feig D.S. (2014). The role of lifestyle interventions in the prevention of gestational diabetes. Curr. Diab. Rep..

[63] Greaves C.J., Sheppard K.E., Abraham C., Hardeman W., Roden M., Evans P.H., Schwarz P., Group I.S. (2011). Systematic review of reviews of intervention components associated with increased effectiveness in dietary and physical activity interventions. BMC Public Health.

[64] Sharma A.K., Singh S., Singh H., Mahajan D., Kolli P., Mandadapu G., Kumar B., Kumar D., Kumar S., Jena M.K. (2022). Deep Insight of the Pathophysiology of Gestational Diabetes Mellitus. Cells.

[65] Buchanan T.A., Xiang A.H., Page K.A., Watanabe R.M. (2025). What Is Gestational Diabetes-Really?. Diabetes.

