# Supplementary material for: Key Components of Prenatal Nutrition Education Interventions to Prevent Gestational Diabetes Mellitus: A Systematic Review and Meta-Analysis
Source: Nutrients. 2026 Oct 8;18(19):3303. doi: 10.3390/nu18193303 (PMC13667000; doi:10.3390/nu18193303)
Supplement: Supplementary file 1 [file nutrients-18-03303-s001.zip › nutrients-4558132-supplementary.pdf]

Table S1. Search strategies used in PubMed, Embase, Web of Science, and the Cochrane Library.

Database	Search Strategy
**PubMed**	(("Diabetes Mellitus"[Mesh] OR "Diabetes, Gestational"[Mesh] OR "Insulin Resistance"[Mesh] OR "Blood Glucose"[Mesh] OR diabetes*[tiab] OR glucose[tiab] OR "gestational diabete*"[tiab] OR "gestational diabetes mellitus"[tiab] OR GDM[tiab] OR "glucose intolerance"[tiab] OR "insulin resistance"[tiab] OR "glycemic control"[tiab] OR "pregnancy-induced diabetes"[tiab]) AND ("Pregnancy"[Mesh] OR pregnan*[tiab] OR gravid*[tiab] OR gestation*[tiab] OR maternal[tiab] OR antenatal[tiab]) AND ((lifestyle[tiab] OR behavior[tiab] OR diet*[tiab] OR nutrition[tiab] OR education[tiab] OR instruction[tiab] OR training[tiab] OR exercise*[tiab] OR "physical activity"[tiab]) AND (intervention[tiab] OR program[tiab] OR modification[tiab])) AND ("Randomized Controlled Trial"[Publication Type] OR "Randomized Controlled Trials as Topic"[Mesh] OR "randomized controlled trial"[tiab] OR random*[tiab]))
**Embase**	(('diabetes mellitus'/exp OR 'gestational diabetes'/exp OR 'insulin resistance'/exp OR 'blood glucose'/exp OR diabetes*:ti,ab OR glucose:ti,ab OR 'gestational diabete*':ti,ab OR 'gestational diabetes mellitus':ti,ab OR gdm:ti,ab OR 'glucose intolerance':ti,ab OR 'insulin resistance':ti,ab OR 'glycemic control':ti,ab OR 'pregnancy-induced diabetes':ti,ab) AND ('pregnancy'/exp OR pregnan*:ti,ab OR gravid*:ti,ab OR gestation*:ti,ab OR maternal:ti,ab OR antenatal:ti,ab) AND ((lifestyle:ti,ab OR behavior:ti,ab OR diet*:ti,ab OR nutrition:ti,ab OR education:ti,ab OR instruction:ti,ab OR training:ti,ab OR exercise*:ti,ab OR 'physical activity':ti,ab) AND (intervention:ti,ab OR program:ti,ab OR modification:ti,ab)) AND ('randomized controlled trial'/exp OR 'randomized controlled trial':ti,ab OR random*:ti,ab))
**Web of Science**	TS=((diabetes* OR glucose OR "gestational diabete*" OR "gestational diabetes mellitus" OR GDM OR "glucose intolerance" OR "insulin resistance" OR "glycemic control" OR "pregnancy-induced diabetes") AND (pregnan* OR gravid* OR gestation* OR maternal OR antenatal) AND ((lifestyle OR behavior OR diet* OR nutrition OR education OR instruction OR training OR exercise* OR "physical activity") AND (intervention OR program OR modification)) AND ("randomized controlled trial" OR random*))
**Cochrane Library**	((diabetes* OR glucose OR "gestational diabete*" OR "gestational diabetes mellitus" OR GDM OR "glucose intolerance" OR "insulin resistance" OR "glycemic control" OR "pregnancy-induced diabetes") AND (pregnan* OR gravid* OR gestation* OR maternal OR antenatal) AND ((lifestyle OR behavior OR diet* OR nutrition OR education OR instruction OR training OR exercise* OR "physical activity") AND (intervention OR program OR modification)) AND ("randomized controlled trial" OR random*)):ti,ab,kw

Table S2. Characteristics of studies included in the meta-analysis of RCTs of prenatal nutrition education interventions to prevent GDM.

Author	Year	Event Intervention	Total Intervention	Event Control	Total Control	Diet Type	Trimester
Bruno R	2016	13	69	23	62	LGI diets	First trimester
Dodd JM	2014	148	1080	120	1073	Balanced diets	Second trimester
Hui A L	2006	1	24	2	21	Balanced diets	Second trimester
Poston L	2013	22	79	24	75	LGI diets	Second trimester
Poston L	2015	160	629	172	651	LGI diets	Second trimester
Melero V	2020	19	128	34	132	LGI diets	First trimester
Garmendia ML	2020	315	1968	181	1395	Balanced diets	First trimester
Zhang Y	2019	45	200	43	200	LGI diets	First trimester
Korpi-Hyövälti E	2011	3	27	1	27	Balanced diets	First trimester
Kunath J	2019	109	1008	106	954	Balanced diets	Second trimester
Koivusalo SB	2016	20	144	27	125	Balanced diets	Second trimester
Walsh JM	2012	7	350	9	371	LGI diets	Second trimester
Wang L	2025	85	245	89	244	Balanced diets	Second trimester
Wang S	2015	23	134	33	138	Balanced diets	First trimester
Xu J	2023	21	128	38	123	Balanced diets	Second trimester
H Al Wattar B	2019	83	377	112	397	Balanced diets	Second trimester
Vinter CA	2014	9	150	8	154	Balanced diets	Second trimester
Harreiter J	2019	44	200	41	201	Balanced diets	Second trimester
Harrison CL	2013	27	121	35	107	Balanced diets	Second trimester

Moses RG	2014	27	296	28	280	LGI diets	Second trimester
Kennelly MA	2018	37	241	36	257	LGI diets	Second trimester
Goodarzi-Khoigani M	2017	1	71	5	71	Balanced diets	First trimester
Luoto R	2011	34	216	22	179	Balanced diets	First trimester
Chan RS	2018	20	80	23	86	Balanced diets	First trimester
Eslami E	2018	15	70	17	70	Balanced diets	Second trimester
Barquiel B	2023	5	85	10	84	Balanced diets	Second trimester
Peacock L	2020	163	640	175	665	LGI diets	Second trimester
Ding B	2021	25	104	42	111	Balanced diets	First trimester
Li CL	2021	4	410	22	410	Balanced diets	First trimester

Table S3. The TIDieR checklist of studies included in the meta-analysis of RCTs of prenatal nutrition education interventions to prevent GDM.

Author	Year	(i) Who	(ii) Tailoring	(iii) Why	(iv) How	(v) What	(vi) Where	(vii) How Much	(viii) How Well
Bruno R	2016	Medical personnel	High	Nutritional theory	Traditional face-to-face	Dietary + physical activity	Hospital	Moderate	Low
Dodd JM	2014	Medical personnel	High	Behavioral theory	Face-to-face + remote	Dietary + physical activity	Hospital	Moderate	High
Hui A L	2006	Medical personnel	High	Nutritional theory	Traditional face-to-face	Dietary + physical activity	Community health center	Moderate	Moderate
Poston L	2013	Research peers	High	Behavioral theory	Face-to-face + remote	Dietary + physical activity	Hospital	Moderate	Moderate
Poston L	2015	Research peers	High	Behavioral theory	Face-to-face + remote	Dietary + physical activity	Hospital	Moderate	Moderate
Melero V	2020	Medical personnel	High	Nutritional theory	Traditional face-to-face	Dietary as main approach	Hospital	Low	High
Garmendia ML	2020	Medical personnel	Low/moderate	Nutritional theory	Traditional face-to-face	Dietary + physical activity	Community health center	Low	Moderate
Zhang Y	2019	Medical personnel	High	Nutritional theory	Face-to-face + remote	Dietary as main approach	Hospital	Low	Moderate
Korpi -Hyövälti E	2011	Medical personnel	High	Nutritional theory	Traditional face-to-face	Dietary as main approach	Community health center	Moderate	Moderate
Kunath J	2019	Medical personnel	Low/moderate	Nutritional theory	Traditional face-to-face	Dietary + physical activity	Community health center	Moderate	Moderate
Koivusalo SB	2016	Medical personnel	High	Nutritional theory	Traditional face-to-face	Dietary + physical activity	Hospital	Low	High

Walsh JM	2012	Medical personnel	High	Nutritional theory	Traditional face-to-face	Dietary as main approach	Hospital	Low	High
Wang L	2025	Medical personnel	High	Nutritional theory	Traditional face-to-face	Dietary as main approach	Hospital	Low	High
Wang S	2015	Medical personnel	Low/moderate	Nutritional theory	Traditional face-to-face	Dietary + physical activity	Hospital	Low	Moderate
Xu J	2023	Medical personnel	High	Nutritional theory	Face-to-face + remote	Dietary + physical activity	Hospital	Low	High
H Al Wattar B	2019	Medical personnel	High	Nutritional theory	Face-to-face + remote	Dietary as main approach	Hospital	Moderate	High
Vinter CA	2014	Medical personnel	Low/moderate	Nutritional theory	Traditional face-to-face	Dietary + physical activity	Hospital	Moderate	Moderate
Harreiter J	2019	Medical personnel	High	Behavioral theory	Face-to-face + remote	Dietary + physical activity	Hospital	Moderate	Moderate
Harrison CL	2013	Medical personnel	High	Behavioral theory	Traditional face-to-face	Dietary + physical activity	Hospital	Low	High
Moses RG	2014	Medical personnel	High	Nutritional theory	Face-to-face + remote	Dietary as main approach	Hospital	Low	High
Kennelly MA	2018	Medical personnel	High	Behavioral theory	Face-to-face + remote	Dietary + physical activity	Hospital	Moderate	Moderate
Goodarzi -Khoigani M	2017	Medical personnel	High	Nutritional theory	Traditional face-to-face	Dietary as main approach	Mixed	Low	Moderate
Luoto R	2011	Medical personnel	High	Nutritional theory	Traditional face-to-face	Dietary + physical activity	Community health center	Moderate	Moderate
Chan RS	2018	Medical personnel	High	Nutritional theory	Face-to-face + remote	Dietary + physical activity	Hospital	Moderate	Moderate

Eslami E	2018	Research peers	Low/moderate	Nutritional theory	Face-to-face + remote	Dietary + physical activity	Community health center	Low	High
Barquiel B	2023	Medical personnel	High	Nutritional theory	Traditional face-to-face	Dietary + physical activity	Hospital	Low	Moderate
Peacock L	2020	Research peers	High	Behavioral theory	Face-to-face + remote	Dietary + physical activity	Hospital	Moderate	Moderate
Ding B	2021	Medical personnel	High	Nutritional theory	Face-to-face + remote	Dietary + physical activity	Hospital	Moderate	High
Li CL	2021	Medical personnel	High	Nutritional theory	Traditional face-to-face	Dietary + physical activity	Hospital	Low	High

Table S4. Sociodemographic characteristics in the 29 included trials.

Study	Race/Ethnicity	Income	Parity (% Nulliparous)
Bruno R, 2016	Caucasian 82.3%; African 12.6%; other 5.2%	NR	Nulliparous 55.2%
Dodd JM, 2014	White 90.5%; Asian 2.7%; Indian 3.4%	SEIFA: 30% most disadvantaged quintile	Nulliparous 40.8%
Hui AL, 2006	Visible minority 61% (mostly Aboriginal)	89% low/low-middle income	NR
Poston L, 2013	White 55%; Black 40%; Asian 2%; other 2%	63% vs 54% most-deprived quintile	Nulliparous 45% vs 43%
Poston L, 2015	White 63%; Black 26%; Asian 6%; other 6%	45% vs 41% most-deprived quintile	Primiparous 43% vs 44%
Melero V, 2020	100% Hispanic	Unemployed 67.8% vs 69.0%	Primiparous 29.4% vs 25.4%
Garmendia ML, 2020	NR	Working 45.2% vs 41.8%	Primipara 45%
Zhang Y, 2019	NR	NR	NR
Korpi-Hyövälti E, 2011	NR	NR	NR
Kunath J, 2019	Born in Germany 88.0% vs 89.5%	NR	Nulliparous 62.0% vs 53.0%
Koivusalo SB, 2016	White	NR	Nulliparous 42% vs 42%
Walsh JM, 2012	NR	NR	0% nulliparous (all secundigravid)
Wang L, 2025	Han 96.2% vs 95.7%	Income >300k CNY: 28.0% vs 34.5%	Primigravida 54.4% vs 59.3%
Wang S, 2015	NR	NR	NR
Xu J, 2023	Han 99.2% vs 99.2%	NR	NR
H Al Wattar B, 2019	White 36.6%; Asian 43.3%; Black 16.4%	NR	Primigravida 27.3%
Vinter CA, 2014	Danish Caucasian	Employed 68.7% vs 68.8%	Primiparous 52.7% vs 54.6%
Harreiter J, 2019	European descent 86.7%	NR	Nulliparous 35.6%
Harrison CL, 2013	Australian-born 38%; S/C Asia 36%; SE Asia 12%	Income <$40k: 30% vs 26%	First pregnancy 42% vs 43%
Moses RG, 2014	NR (recorded, not tabulated)	NR	Parity 0.8 (SEM 0.1) in both

Kennelly MA, 2018	Caucasian 93.2% vs 89.2%	NR	Nulliparous 49.6% vs 53.7%
Goodarzi-Khoigani M, 2017	Iranian (eligibility criterion)	Income >12M rials: 29.6% vs 18.3%	100% primiparous
Luoto R, 2011	NR	NR	Primiparous 47.0% vs 40.6%
Chan RS, 2018	100% Chinese	Family income ≥HK$20,000: 83.8% vs 69.8%	Nulliparous 61.2% vs 58.1%
Eslami E, 2018	NR	Adequate income 20.0% vs 11.4%	NR
Barquiel B, 2023	Caucasian 64.7% vs 68.7%; Latin American 30.6% vs 28.9%	NR	Median pregnancies 2
Peacock L, 2020	White 60.5%–65.4%; Black 23.8%–28.5%; Asian 5.3%–6.9%	40.4%–51.6% most-deprived quintile	Nulliparous 39.9%–44.8%
Ding B, 2021	100% Chinese	NR	Primiparous 72.0%
Li CL, 2021	NR	NR	Nulliparous 71% vs 70%

Abbreviations: NA, not applicable; NR, not reported.

Table S5. Univariable random-effects meta-regressions of study-level characteristics on GDM risk.

`	k	β (95% CI)	exp(β)	P value	R^2, %
Mean maternal age, per year	28	-0.070 (-0.110 to -0.031)	0.93	<0.001	72.7
Mean pre-pregnancy BMI, per kg/m²	26	0.005 (-0.022 to 0.032)	1.00	0.74	0.0
Nulliparous women, per 10%	21	-0.048 (-0.146 to 0.050)	0.95	0.33	0.0
Enrollment restricted to obesity (yes vs no)	29	0.106 (-0.154 to 0.366)	1.11	0.42	0.0

Meta-regressions used restricted maximum likelihood estimation.

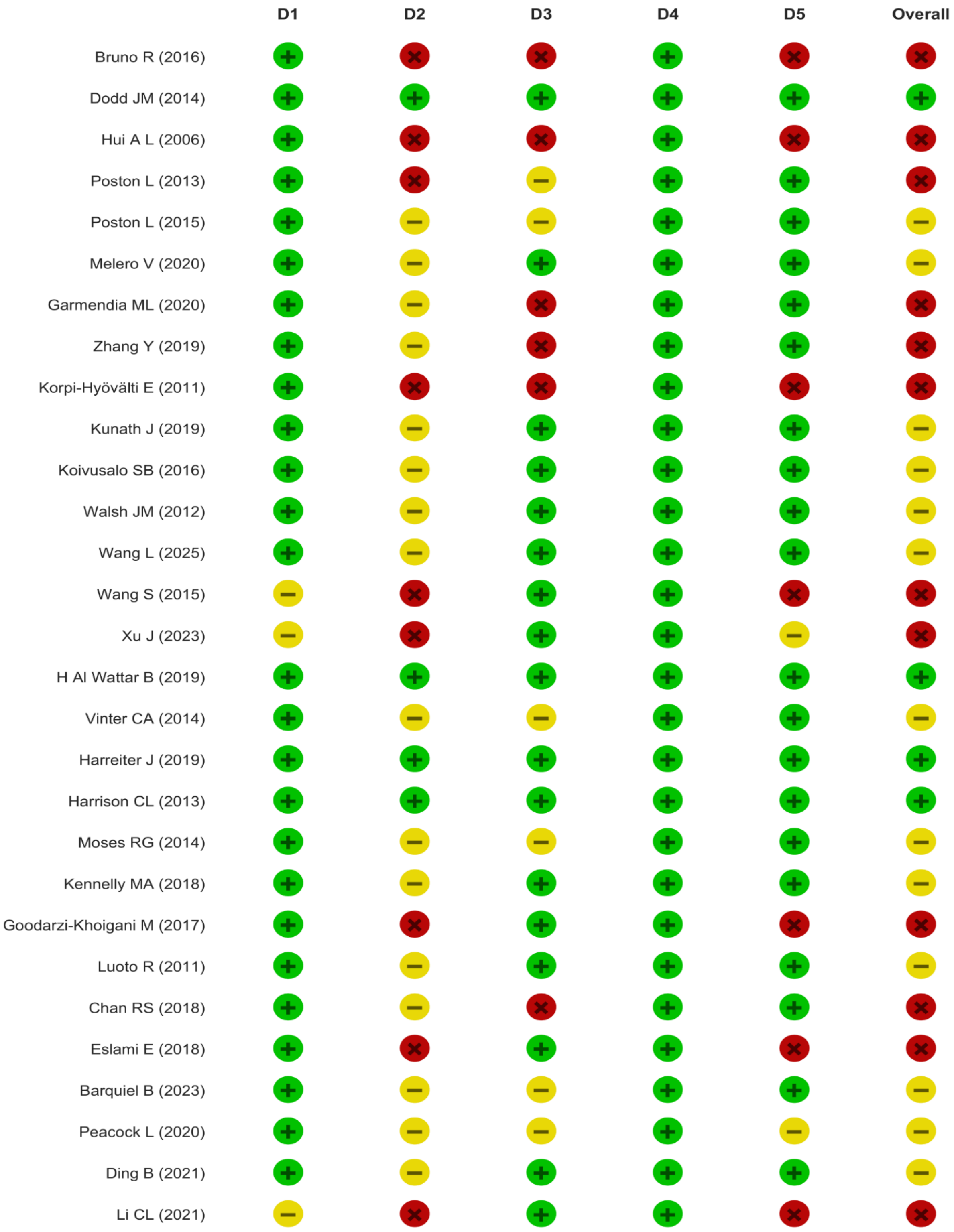

Figure S1. Traffic light plot summarizing the risk-of-bias judgments across included studies according to ROB 2.0.

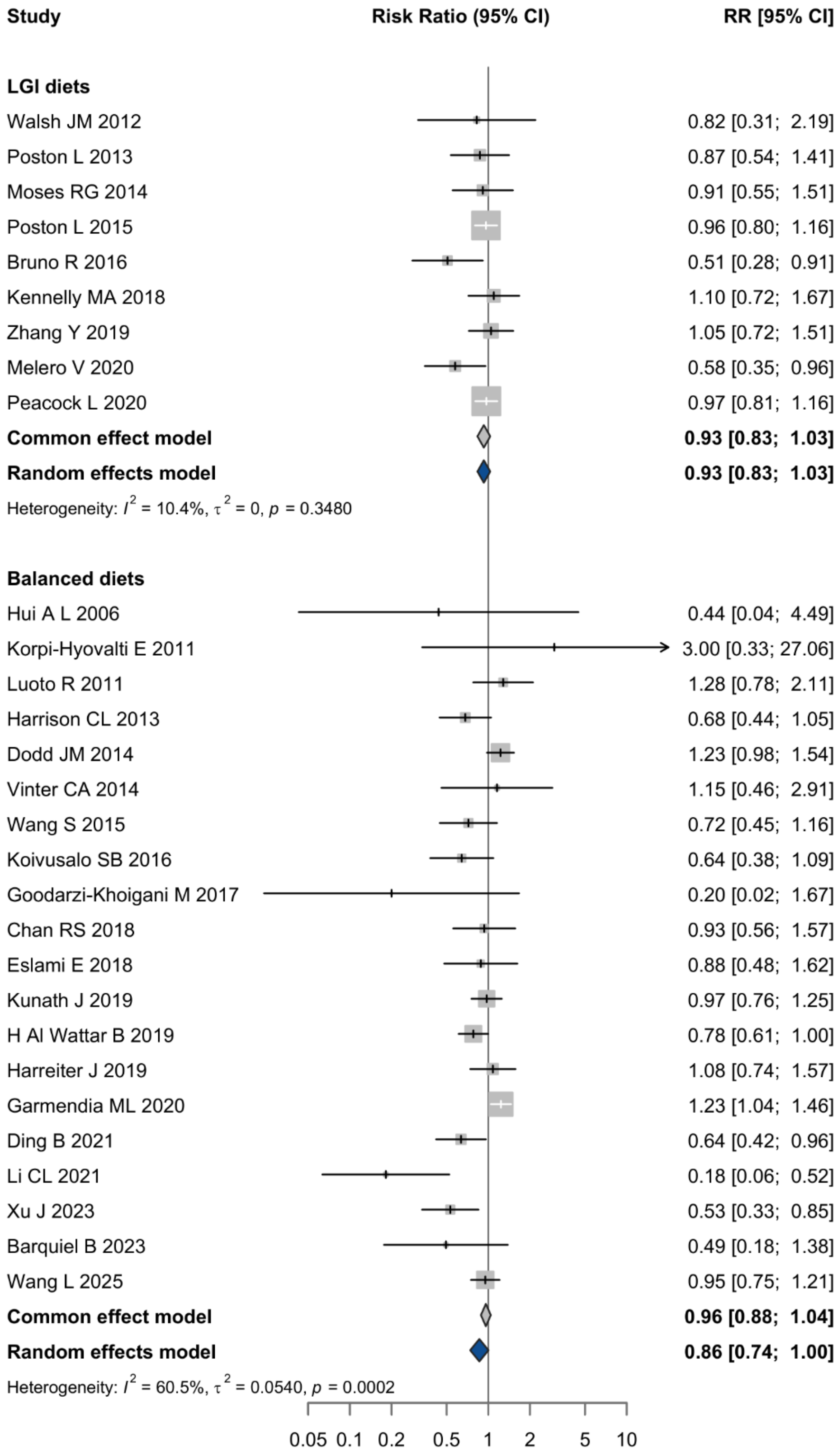

Figure S2. Meta-analysis of subgroup analyses stratified by dietary intervention type.

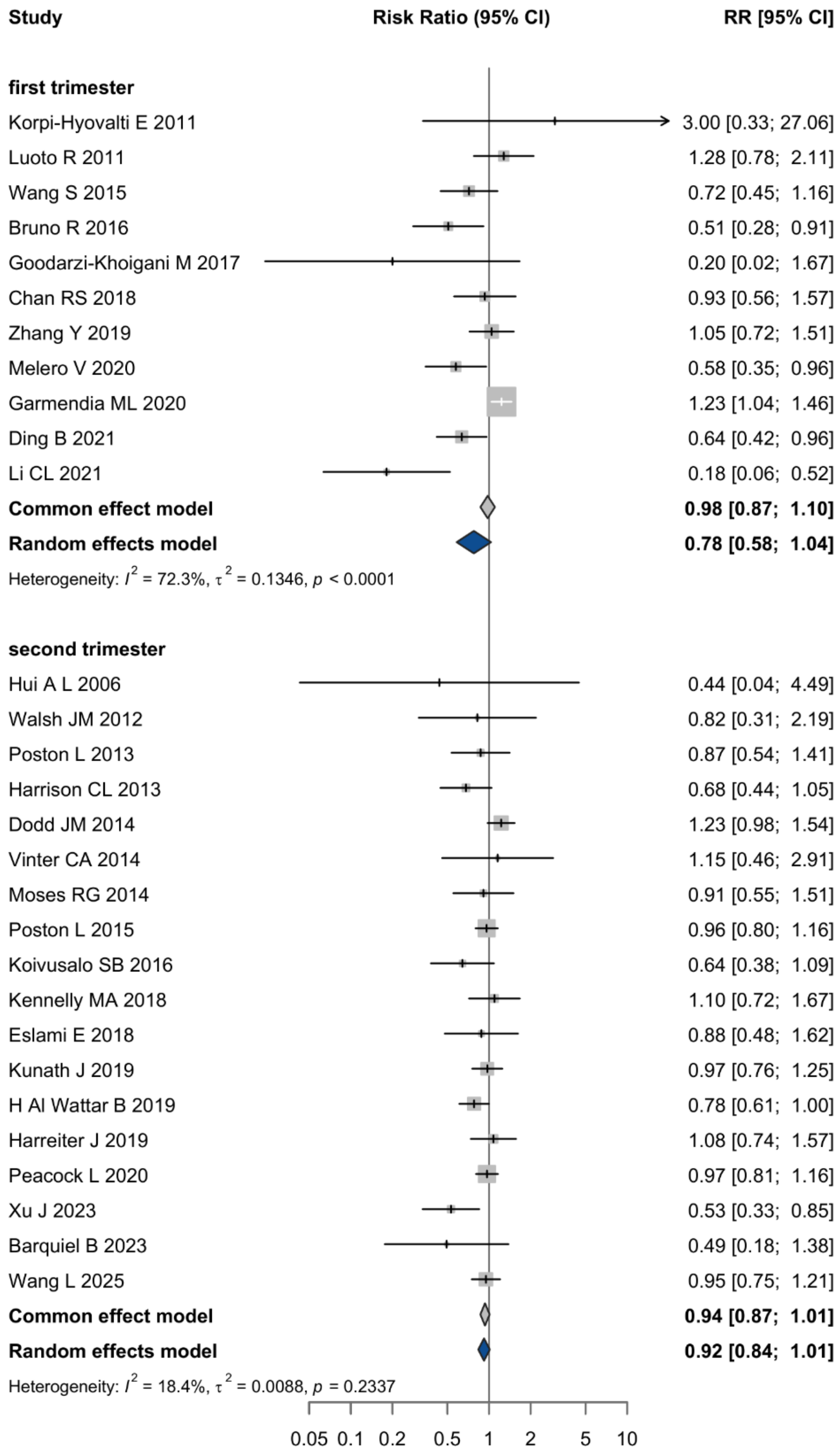

Figure S3. Meta-analysis of subgroup analyses stratified by trimester of intervention initiation.

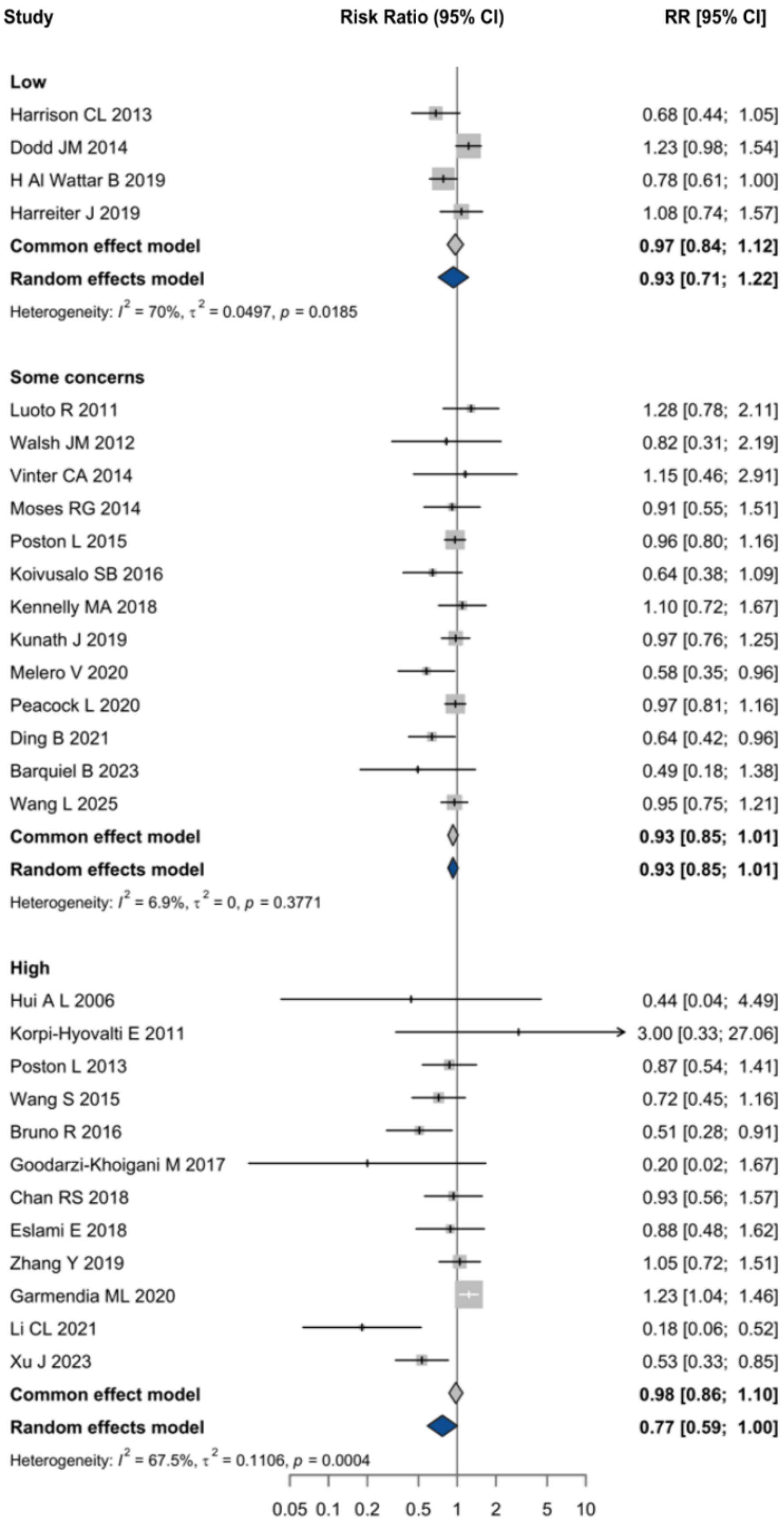

Figure S4. Meta-analysis of subgroup analyses stratified by risk of bias.

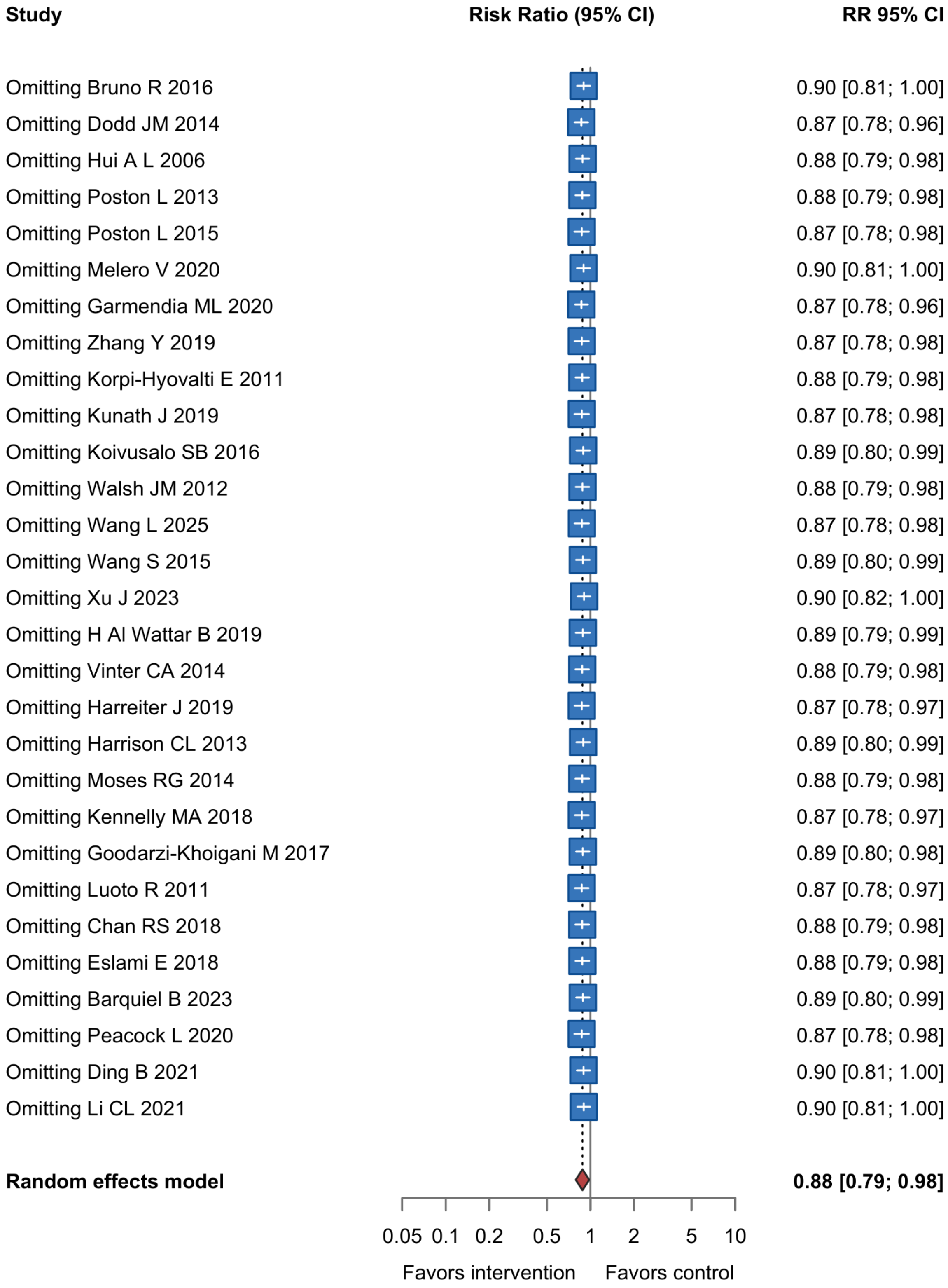

Figure S5. The leave-one-out sensitivity analysis of included RCTs.

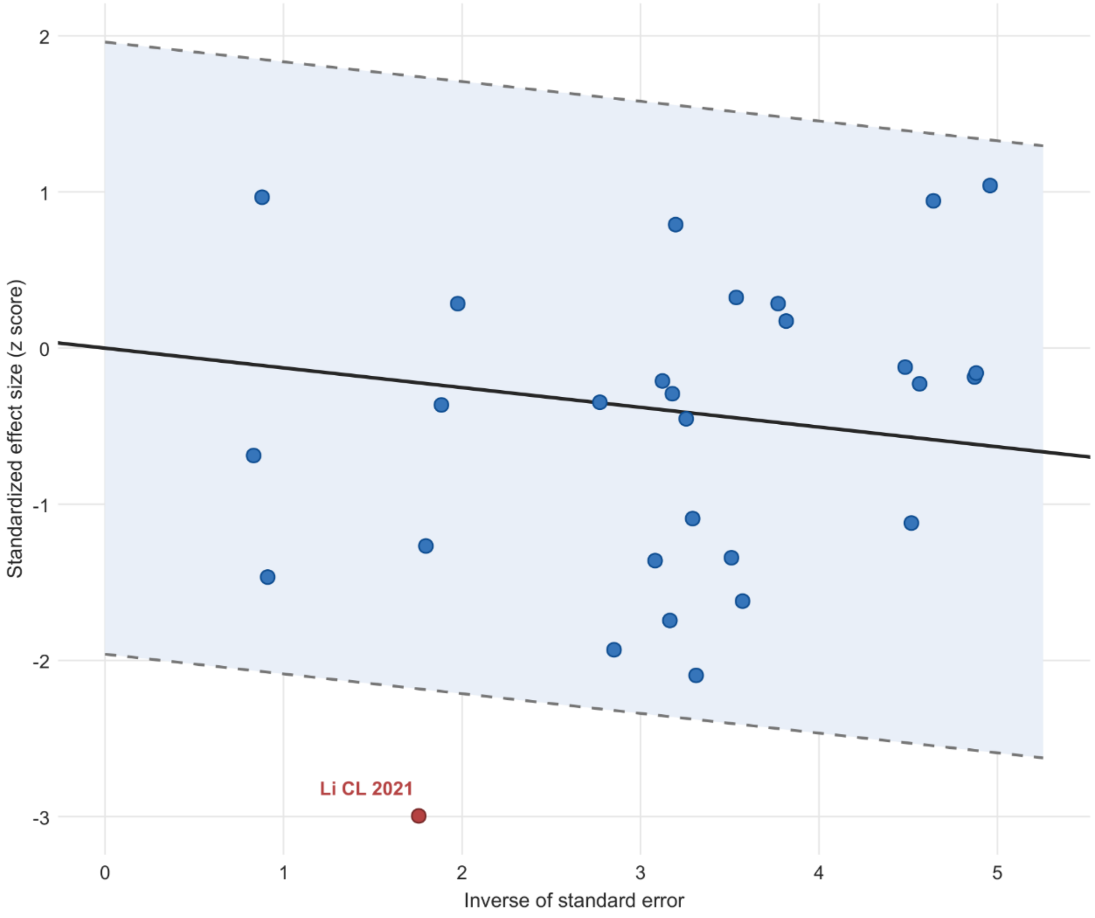

Figure S6. The Galbraith plot of included RCTs. The solid line represents the pooled random-effects estimate; the dashed lines mark the 95% confidence bounds ($z = \pm 1.96$). One study (Li CL, 2021) fell outside these bounds and was identified as a statistical outlier ($z = -3.00$).

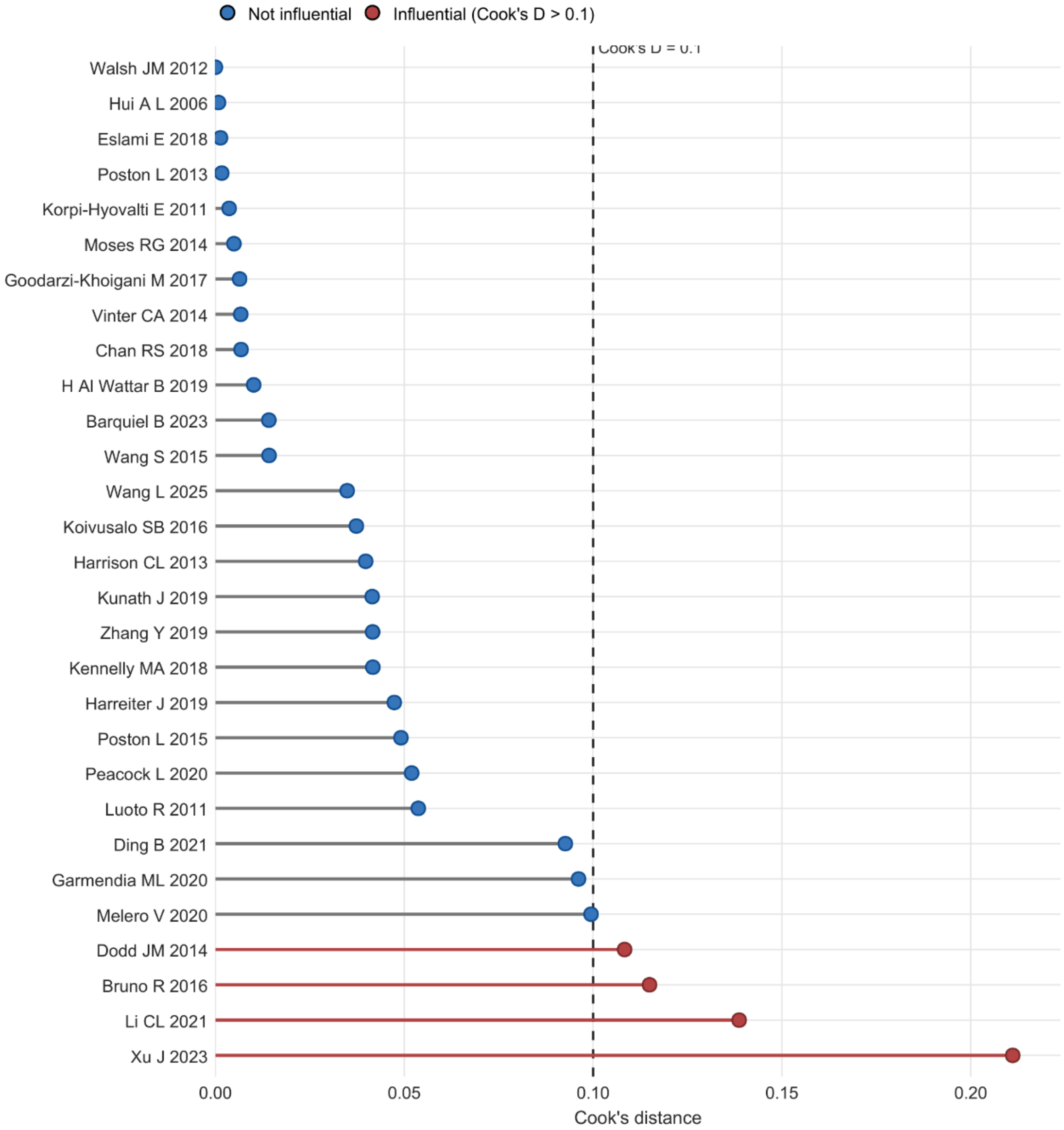

Figure S7. Cook's distances for each included study in the random-effects meta-analysis. The dashed vertical line marks the threshold of 0.1; studies exceeding this threshold (red) were considered potentially influential.

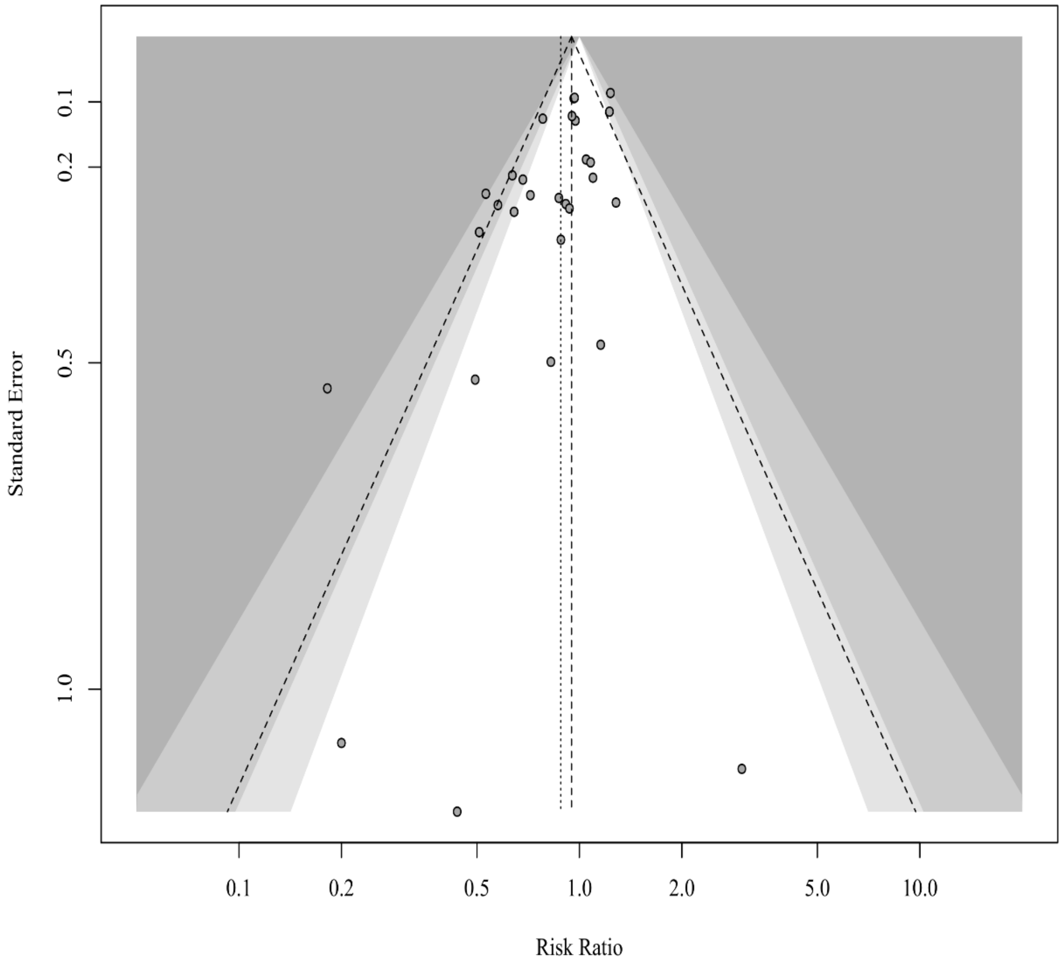

Figure S8. The contour-enhanced funnel plot of included RCTs.

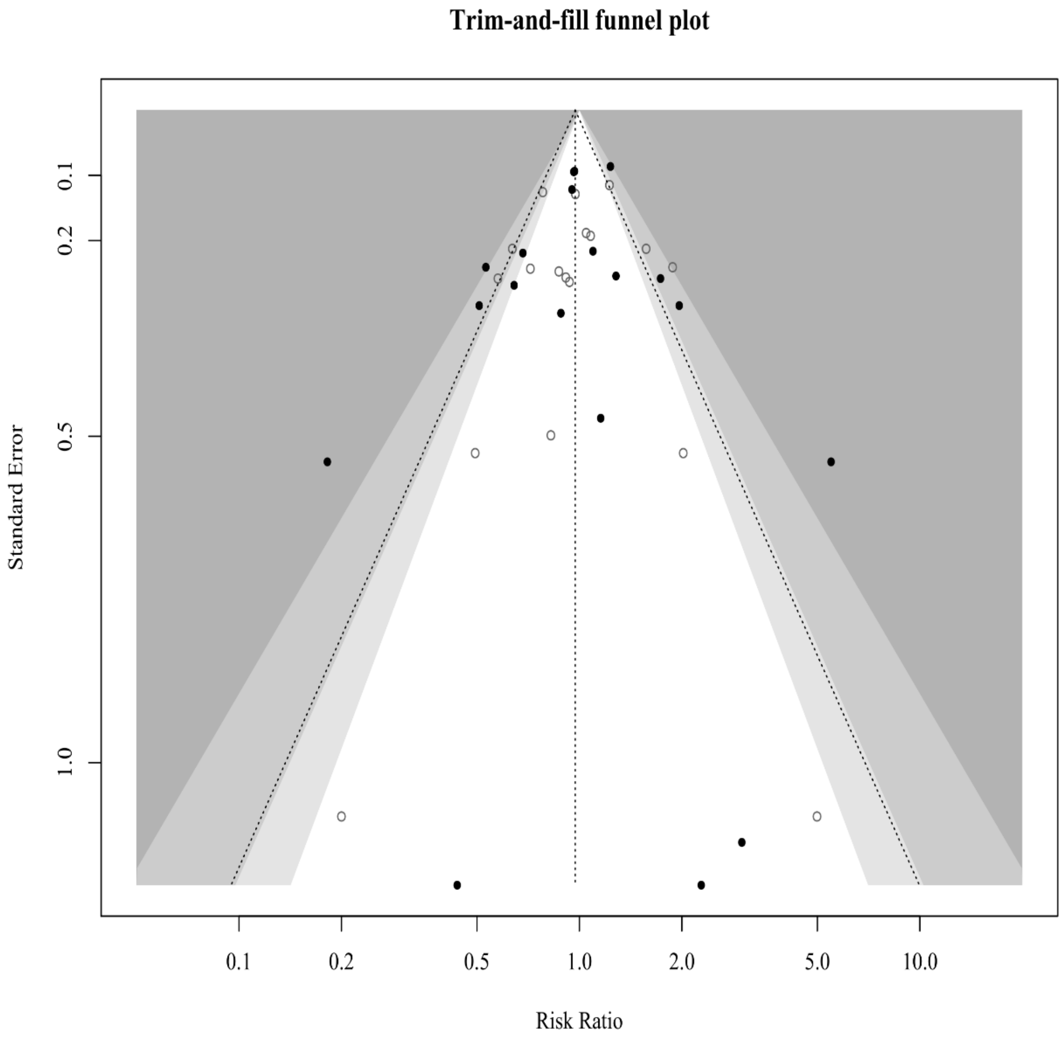

Figure S9. The trim-and-fill funnel plot of included RCTs.
